# Activation of the *Plasmodium* Egress Effector Subtilisin-Like Protease 1 Is Mediated by Plasmepsin X Destruction of the Prodomain

**DOI:** 10.1128/mbio.00673-23

**Published:** 2023-04-10

**Authors:** Sumit Mukherjee, Armiyaw S. Nasamu, Kelly C. Rubiano, Daniel E. Goldberg

**Affiliations:** a Division of Infectious Diseases, Department of Medicine, and Department of Molecular Microbiology, Washington University School of Medicine, St. Louis, Missouri, USA; Stanford University

**Keywords:** aspartic protease, egress, malaria

## Abstract

Following each round of replication, daughter merozoites of the malaria parasite Plasmodium falciparum escape (egress) from the infected host red blood cell (RBC) by rupturing the parasitophorous vacuole membrane (PVM) and the RBC membrane (RBCM). A proteolytic cascade orchestrated by a parasite serine protease, subtilisin-like protease 1 (SUB1), regulates the membrane breakdown. SUB1 activation involves primary autoprocessing of the 82-kDa zymogen to a 54-kDa (p54) intermediate that remains bound to its inhibitory propiece (p31) postcleavage. A second processing step converts p54 to the terminal 47-kDa (p47) form of SUB1. Although the aspartic protease plasmepsin X (PM X) has been implicated in the activation of SUB1, the mechanism remains unknown. Here, we show that upon knockdown of PM X, the inhibitory p31-p54 complex of SUB1 accumulates in the parasites. Using recombinant PM X and SUB1, we show that PM X can directly cleave both p31 and p54. We have mapped the cleavage sites on recombinant p31. Furthermore, we demonstrate that the conversion of p54 to p47 can be effected by cleavage at either SUB1 or PM X cleavage sites that are adjacent to one another. Importantly, once the p31 is removed, p54 is fully functional inside the parasites, suggesting that the conversion to p47 is dispensable for SUB1 activity. Relief of propiece inhibition via a heterologous protease is a novel mechanism for subtilisin activation.

## INTRODUCTION

Malaria remains the most significant parasitic disease throughout the world in terms of morbidity and mortality, with Plasmodium falciparum being the deadliest species (1). During the symptomatic blood stage of infection, the parasites replicate by schizogony within a membrane-enclosed parasitophorous vacuole (PV) inside the host red blood cells (RBCs). Following each round of replication, the daughter merozoites escape from the infected RBCs by rupturing the parasitophorous vacuole membrane (PVM) and RBC membrane (RBCM) through an explosive process called egress (2). As egress allows the released merozoites to invade fresh RBCs, it is critical for the parasite’s dissemination.

Parasite egress follows an “inside-out” route where the PVM disruption precedes the RBCM rupture (3, 4). Upon schizont maturation and approximately 10 min prior to the actual release of the merozoites, the PVM first rounds up due to an intracellular calcium oscillation (2). Calcium also activates the cyclin-dependent protein kinase 5 (CDPK5) (5). Within the next few minutes, the parasite’s cGMP-dependent protein kinase G (PKG) is activated. In coordination with CDPK5, the activated PKG triggers discharge of the subtilisin-like serine protease 1 (SUB1) from the exonemes into the PV (6–8). PKG-mediated SUB1 discharge is a prerequisite for PVM rupture (9). Once inside the PV, SUB1 proteolytically activates several merozoite surface proteins and soluble proteases, including the merozoite surface proteins (MSPs) and SERA6, respectively (9, 10). The coordinated action of these effectors leads to the disassembly of the host cell cytoskeleton, perforation, and, finally, rupture of the RBCM.

SUB1 is synthesized as a precursor of 82 kDa (p82) with a C-terminal catalytic domain and an N-terminal extension called the prodomain (PD) (11). Following translocation into the endoplasmic reticulum (ER), p82 undergoes autocatalytic cleavage to yield a C-terminal 54-kDa intermediate (p54) that remains noncovalently bound to the cleaved PD, also called the propiece (p31) (11). Post-ER but before discharge, p54 is further processed to a 47-kDa form (p47). In parasites, this second cleavage of SUB1 is dependent on the exoneme-localized aspartic protease plasmepsin X (PM X) (12, 13). Importantly, transgenic parasites lacking PM X or parasites treated with PM X inhibitors fail to process SUB1 substrates, leading to an egress block (12–15). This suggests that PM X is involved in the activation of SUB1, although the mechanism remains unknown.

Before SUB1 can be activated, the PD needs to be removed. For most subtilisins, the PD is required for folding and is inhibitory until removal (16). In the apicomplexan Toxoplasma gondii, the microneme protein TgMIC5 acts as a PD for TgSUB1 and regulates the enzymatic activity of the latter (17, 18). TgMIC5 shares both structural and sequence similarities with the PfSUB1 PD and the PDs from other *Plasmodium* species (17). This hints at a similar regulatory role for the PfSUB1 PD in the activity of the protease. In fact, a recombinantly made p31 was shown to inhibit the activity of both the recombinant and native SUB1 (19). Consistent with its inhibitory function, crystal structures of SUB1 revealed a tight interaction between p31 and the catalytic domain, occluding the active site (20, 21).

In this study, we sought to understand the following mechanistic questions. How does the knockdown of PM X lead to the accumulation of the inactive form of SUB1? Does conversion of p54 to p47 activate SUB1? We report that PM X removes the inhibitory p31 from the p31-p54 complex by directly cleaving p31 at multiple sites. We further show that once p31 is removed by proteolytic cleavage, the p54 form is fully functional inside the parasites. The involvement of an upstream protease in the removal of the propiece is a novel mechanism for subtilisin activation.

## RESULTS

### PM X mediates cleavage of p31 in the p31-p54 complex of SUB1.

**FIG 1:**
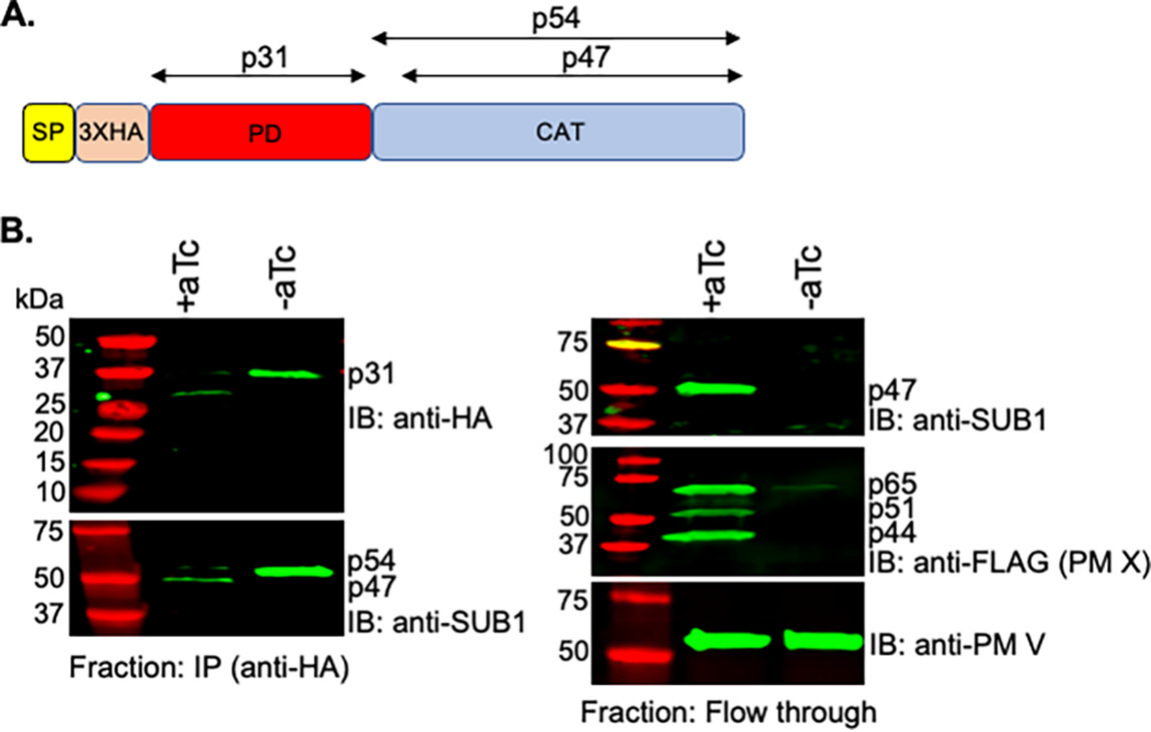
PM X mediates cleavage of SUB1 p31 *in vivo*. (A) Schematic of the modified endogenous SUB1 in the aTc-regulatable PM X^apt^ strain of P. falciparum. The triple hemagglutinin (3×HA) tag was placed between the signal peptide (SP) and the prodomain (PD), thereby enabling us to detect p31 by Western blotting. CAT, catalytic domain. (B) Western blotting. MACS-synchronized PM X^apt^ parasites with edited SUB1 were grown in the presence or absence of aTc for 45 h. Cell lysates prepared from parasite samples were subjected to immunoprecipitation with anti-HA antibodies. The immunoprecipitated fractions (IPs) were fractionated by SDS-PAGE and blotted with the indicated antibodies (left). Western blot analysis was also performed with the flowthrough fractions to detect the C-terminal domain of SUB1 (right, top), PM X (right, middle), and PM V (loading control, right, bottom). IB, immunoblot. Experiments were repeated three times, and shown is a representative blot.

Existing antibodies against SUB1 can detect the p54 and p47 species but not p31 (6). Therefore, to visualize p31 in cultured parasites, we tagged the N terminus of SUB1 with triple hemagglutinin (3×HA). To accomplish this, we replaced the endogenous locus with a recodonized version of *Sub1* by CRISPR/Cas9-mediated genome editing. In the recodonized *Sub1*, the 3×HA was inserted between the predicted signal peptide sequence and the start of p31, thus labeling p31 at its N-terminal end (Fig. 1A). We edited the *Sub1* locus in the anhydrotetracycline (aTc)-regulated PM X^apt^ transgenic parasite line (12) to track the fate of p31 in the presence or absence of PM X. As shown in Fig. 1B (left, top), we were able to pull down p31 from parasite lysates in the absence of the PM X. Additionally, we observed that the p54 coprecipitated with p31 nearly completely under the PM X knockdown condition (Fig. 1B, left, bottom). This validated the physical association between p54 and p31, as reported previously (11). In contrast, p47 was the major species of SUB1 in parasites expressing PM X and was overwhelmingly detected in the flowthrough fraction (Fig. 1, right, top). Together, these data suggest that PM X mediates the degradation of p31, thereby destabilizing the p31-p54 complex of SUB1.

### Recombinant PM X cleaves recombinant p31 *in vitro* at multiple sites.

**FIG 2:**
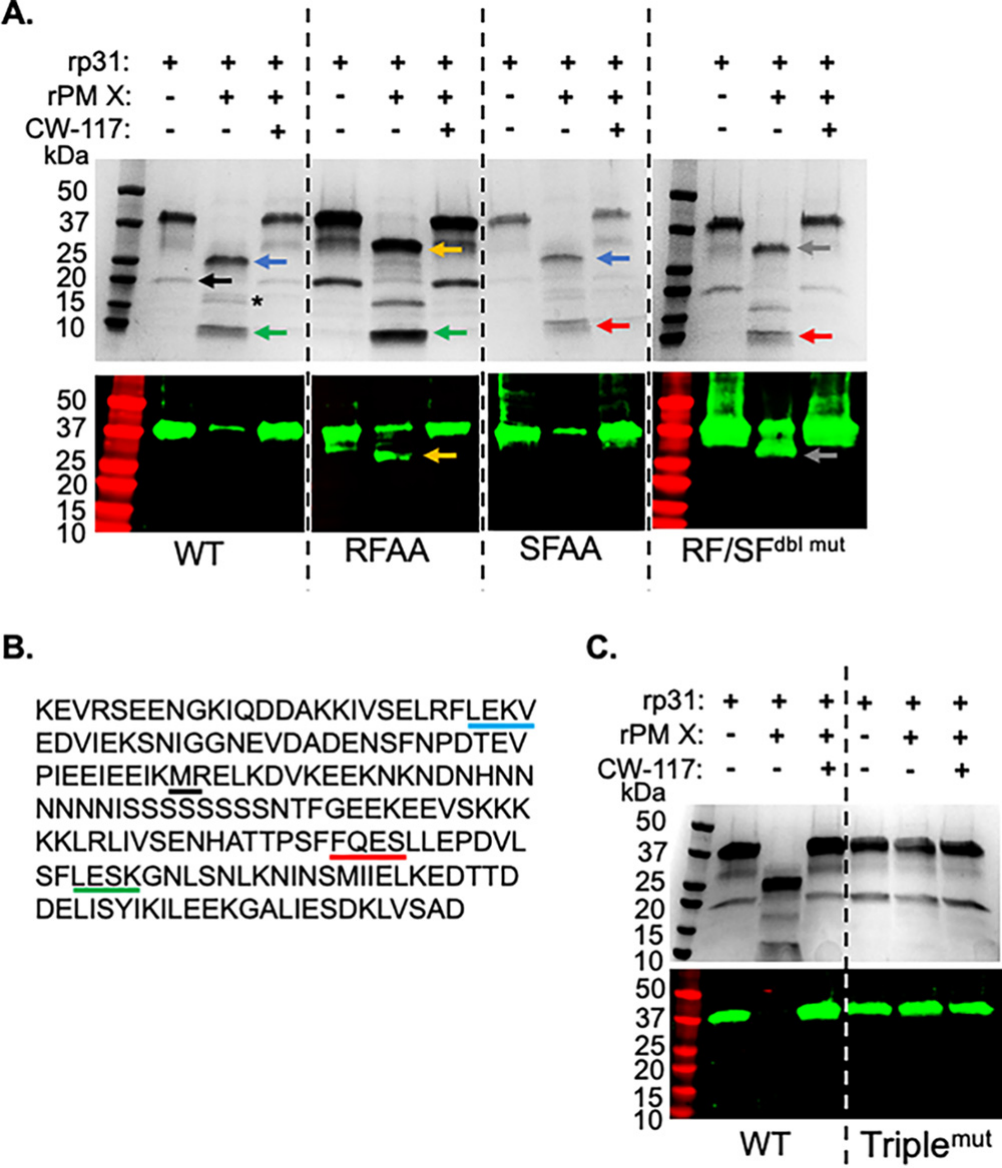
PM X cleaves p31 *in vitro* at alternate sites. (A) Coomassie blotting (top) and anti-HA antibody Western blotting (bottom) showing the cleavage of recombinant p31 (rp31), either wild type (WT) or the indicated mutants, by recombinant PM X (rPM X) following a 3-h assay in the PM X activity buffer. As shown, addition of the PM X inhibitor CWHM-117 (CW-117) inhibited PM X-mediated cleavage of p31. Blue arrows, cleavage product with an N-terminal tetrapeptide LEKV; green arrows, cleavage product with an N-terminal tetrapeptide LESK; orange arrows, N-terminal cleavage product in the RFAA mutant; gray arrow, N-terminal cleavage product in the RFAA/SFAA double mutant (RF/SF^dbl mut^); red arrows, cleavage product with an N-terminal tetrapeptide FQES; black arrow and star, secondary polypeptide translated from an internal methionine and its cleavage product, respectively. (B) Sequence of SUB1 p31 with the N-terminal tetrapeptide sequences for the cleaved p31 fragments and the internal start codon for secondary translated polypeptide in panel A underlined. Colors correspond to the arrows highlighted by the same colors as in panel A. (C) Coomassie blotting (top) and Western blotting with anti-HA antibody (bottom) showing that a triple cleavage mutant p31 (Triple^mut^) is resistant to cleavage by rPM X. Experiments were repeated three times, and shown are representative blots.

To further understand the action of PM X on p31 of SUB1, we carried out an *in vitro* cleavage assay using recombinantly synthesized proteins. Recombinant PM X (rPM X) was produced from mammalian cells as reported earlier (13). For recombinant p31 (rp31), the p31 sequence was expressed and purified from Escherichia coli. This rp31 was tagged at the N- and C-terminal ends with 3×HA and 6 histidines (6×His), respectively. As shown by Coomassie staining of an SDS-PAGE and by Western blotting with an anti-HA antibody (Fig. 2A, wild type [WT]), the full-length form of purified rp31 ran close to the 37-kDa-molecular-weight marker. Additionally, a minor translation product of about 20 kDa was seen only by Coomassie staining (Fig. 2A, black arrow). N-terminal sequencing of this band by Edman degradation confirmed that it was translated from an internal start codon (Fig. 2B, met^63^, black underline; see Data Set S1 in the supplemental material). As revealed by both Coomassie staining and Western blotting, the 37-kDa band was significantly reduced when the rp31 was treated with rPM X (Fig. 2A, WT). In addition, we observed two extra bands of approximately 25 kDa and 10 kDa by Coomassie blotting but not by Western blotting with the anti-HA antibody (Fig. 2A, blue and green arrows, respectively), indicating that both bands were C-terminal fragments. Finally, addition of the PM X inhibitor CWHM-117^12^ into the reaction mixture blocked the processing of rp31 to these shorter fragments. Together, these data suggest that the rPM X is able to cleave rp31 at two sites, of which one is close to the N terminus.

10.1128/mbio.00673-23.6 DATA SET S1 N-terminal sequencing for data associated with Fig. 2. Download Data Set S1, PDF file, 0.5 MB. Copyright © 2023 Mukherjee et al. 2023 Mukherjee et al. https://creativecommons.org/licenses/by/4.0/ This content is distributed under the terms of the Creative Commons Attribution 4.0 International license.

To precisely determine the PM X cleavage sites on rp31, we excised the 25-kDa and 10-kDa bands from SDS-PAGE and subjected them to N-terminal sequencing. For both fragments, unique profiles of residues were observed during the first four cycles of Edman degradation. The N-terminal tetrapeptide sequence for the 25-kDa fragment was LEKV (Fig. 2B, blue underline; Data Set S2), while that for the 10-kDa fragment was LESK (Fig. 2B, green underline; Data Set S3). Of note, the amino acid sequences that span the N-terminal ends of these fragments (Fig. 2B, ^48^RFLE^51^ and ^164^SFLE^167^) conform with the known PM X substrate cleavage specificity (15). To test this further, we expressed two different mutant rp31 versions in E. coli by mutating the last two residues within the ^48^RFLE^51^ or ^164^SFLE^167^ motifs to alanine (^48^RFLE to ^48^RFAA or ^164^SFLE to ^164^SFAA). Such dimutation was shown to abolish PM X cleavage of a fluorogenic peptide or self-processing (13–15). When treated with rPM X, the RFAA mutant was processed to generate two fragments, one running at 10 kDa similar to that in the WT and the other running at approximately 27 kDa (Fig. 2A, green and orange arrows, respectively). Importantly, the 27-kDa fragment retained the N-terminal 3×HA tag and was detected by Western blotting with anti-HA antibody. Interestingly, when treated with rPM X, the processed fragments from ^164^SFAA mutant rp31 ran similarly to those from WT rp31. N-terminal sequencing of the shorter fragment (Fig. 2A, red arrow), however, revealed a different tetrapeptide (FQES in Fig. 2B, red underline; Data Set S4) that is just 9 amino acids upstream of the ^164^SFLE^167^ sequence. Mutating both ^48^RFLE^51^ and ^164^SFLE^167^ sequences together (^48^RF-^164^SF^dbl mut^), on the other hand, generated a PM X cleavage pattern with an intact N-terminal fragment of about 27 kDa (Fig. 2A, gray arrow) and a C-terminal 10-kDa (Fig. 2A, single star) fragment with an N-terminal tetrapeptide, FQES. This suggested that rPM X could cleave both the ^164^SFAA and the ^48^RF-^164^SF^dbl mut^ at the same site, generating identical C-terminal fragments. Consistent with this, the amino acids flanking the N terminus of this fragment (^151^SFFQ^154^) (Fig. 2B) also conform with the PM X cleavage specificity. To check this further, we made a triple mutant rp31 by mutating the ^48^RFLE^51^, ^151^SFFQ^154^, and ^164^SFLE^167^ sites together (Triple^mut^). As shown by both Coomassie staining and Western blotting, Triple^mut^ rp31 was completely resistant to rPM X-mediated cleavage (Fig. 2C). Together, the data suggest that the rPM X can directly cleave rp31 at multiple sites.

10.1128/mbio.00673-23.7 DATA SET S2 N-terminal sequencing for data associated with Fig. 2. Download Data Set S2, PDF file, 0.5 MB. Copyright © 2023 Mukherjee et al. 2023 Mukherjee et al. https://creativecommons.org/licenses/by/4.0/ This content is distributed under the terms of the Creative Commons Attribution 4.0 International license.

10.1128/mbio.00673-23.8 DATA SET S3 N-terminal sequencing for data associated with Fig. 2. Download Data Set S3, PDF file, 0.5 MB. Copyright © 2023 Mukherjee et al. 2023 Mukherjee et al. https://creativecommons.org/licenses/by/4.0/ This content is distributed under the terms of the Creative Commons Attribution 4.0 International license.

10.1128/mbio.00673-23.9 DATA SET S4 N-terminal sequencing for data associated with Fig. 2. Download Data Set S4, PDF file, 0.5 MB. Copyright © 2023 Mukherjee et al. 2023 Mukherjee et al. https://creativecommons.org/licenses/by/4.0/ This content is distributed under the terms of the Creative Commons Attribution 4.0 International license.

**FIG 3:**
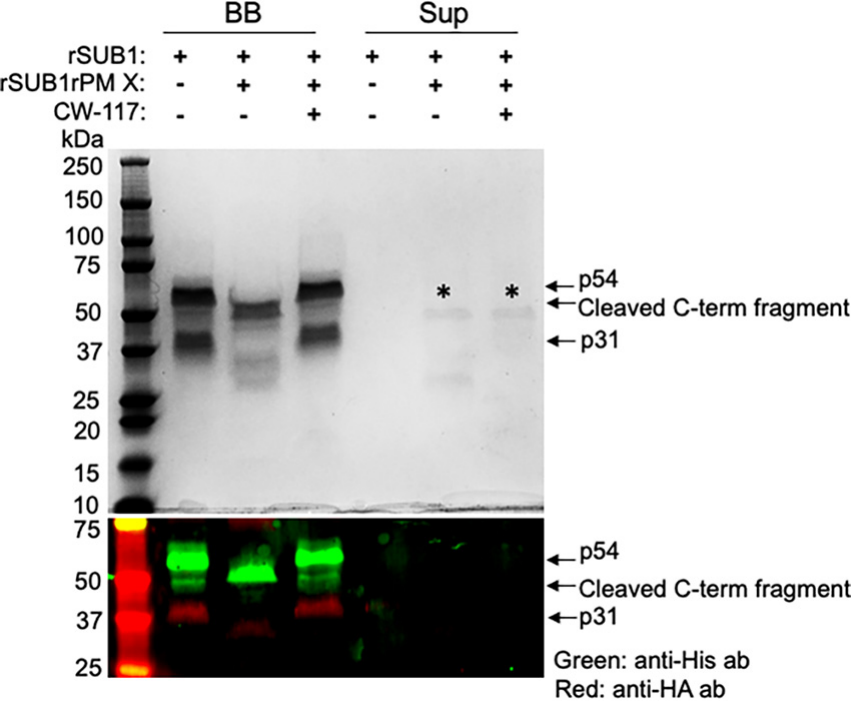
PM X cleaves both p31 and p54 of SUB1 *in vitro*. Coomassie blotting (top) and Western blotting with indicated antibodies (bottom) showing the cleavage of p31 and the C-terminal p54 fragment from the p31-p54 complex of a mammalian expressed recombinant SUB1 (rSUB1). The rSUB1 was tagged at the N- and C-terminal ends with 3×HA and 6×His, respectively. The purified p31-p54 complex was immobilized on HisTrap magnetic beads, and a PM X cleavage assay was performed on the beads with rPM X in the PM X activity buffer. Three hours postincubation, the supernatants (Sup) were separated by placing the reaction tubes on a magnet. The C-terminal fragments of rSUB1 that remained bead bound (BB) were eluted with buffer containing 500 mM imidazole. Both the Sup and BB fractions were boiled in SDS-PAGE buffer and analyzed. Stars indicate PM X. The experiment was repeated three times, and shown are representative blots.

We were also able to purify a recombinant form of SUB1 (rSUB1) from mammalian cells as a secreted protein (Fig. S1). This rSUB1 was tagged at the N and C termini with 3×HA and 6×His, respectively. As reported earlier (11), in the presence of tunicamycin added to block N-glycosylation, purified rSUB1 was a complex of the processed p31-p54. When the p31-p54 complex was incubated in PM X activity buffer, only in the presence of rPM X, we observed cleavage of p31 by Coomassie staining and Western blotting (Fig. 3). This suggested that the rPM X can access the target sites on p31 in the correctly folded rSUB1. Consistent with this, by molecular dynamics analysis, we found that the PM X cleavage sites modeled on P. falciparum SUB1 (*Pv*SUB1) (21) are surface exposed (Fig. S2). Additionally, in the rPM X-treated sample, we observed that the C-terminal fragment ran faster than that in the control sample (no PM X), indicating that rPM X-mediated processing of the p54 fragment occurred *in vitro.*

10.1128/mbio.00673-23.1 FIG S1 Purified recombinant SUB1 (rSUB1) from mammalian Expi293 cells. As described in Materials and Methods, rSUB1, which was tagged at the N- and C-terminal ends with 3×HA and 6×His tags respectively, and was purified as a secreted protein. Treatment of the transfected cells with tunicamycin resulted in the correct folding of rSUB1, which was then secreted mostly as the semiprocessed p31-p54 complex. Download FIG S1, TIF file, 2.0 MB. Copyright © 2023 Mukherjee et al. 2023 Mukherjee et al. https://creativecommons.org/licenses/by/4.0/ This content is distributed under the terms of the Creative Commons Attribution 4.0 International license.

10.1128/mbio.00673-23.2 FIG S2 Crystal structure of SUB1 from Plasmodium vivax. Shown is a ribbon structure with the prodomain (light blue), and the catalytic domain (green). The catalytic triad (D-H-S) is highlighted as red, orange, and pink spheres. Equivalent motifs for the PM X cleavage sites on the prodomain of *Pf*SUB1 (^48^RFLQ, ^135^SFFE, and ^148^SFIQ) are highlighted as dark blue spheres. The figure was constructed from PDB accession number 4TR2 by using PyMOL Molecular Graphics System, version 2.3. The unstructured part of the prodomain was excluded. Download FIG S2, TIF file, 2.0 MB. Copyright © 2023 Mukherjee et al. 2023 Mukherjee et al. https://creativecommons.org/licenses/by/4.0/ This content is distributed under the terms of the Creative Commons Attribution 4.0 International license.

### p54-to-p47 conversion involves both direct cleavage by PM X and SUB1 autocatalysis but is dispensable for parasite growth.

**FIG 4:**
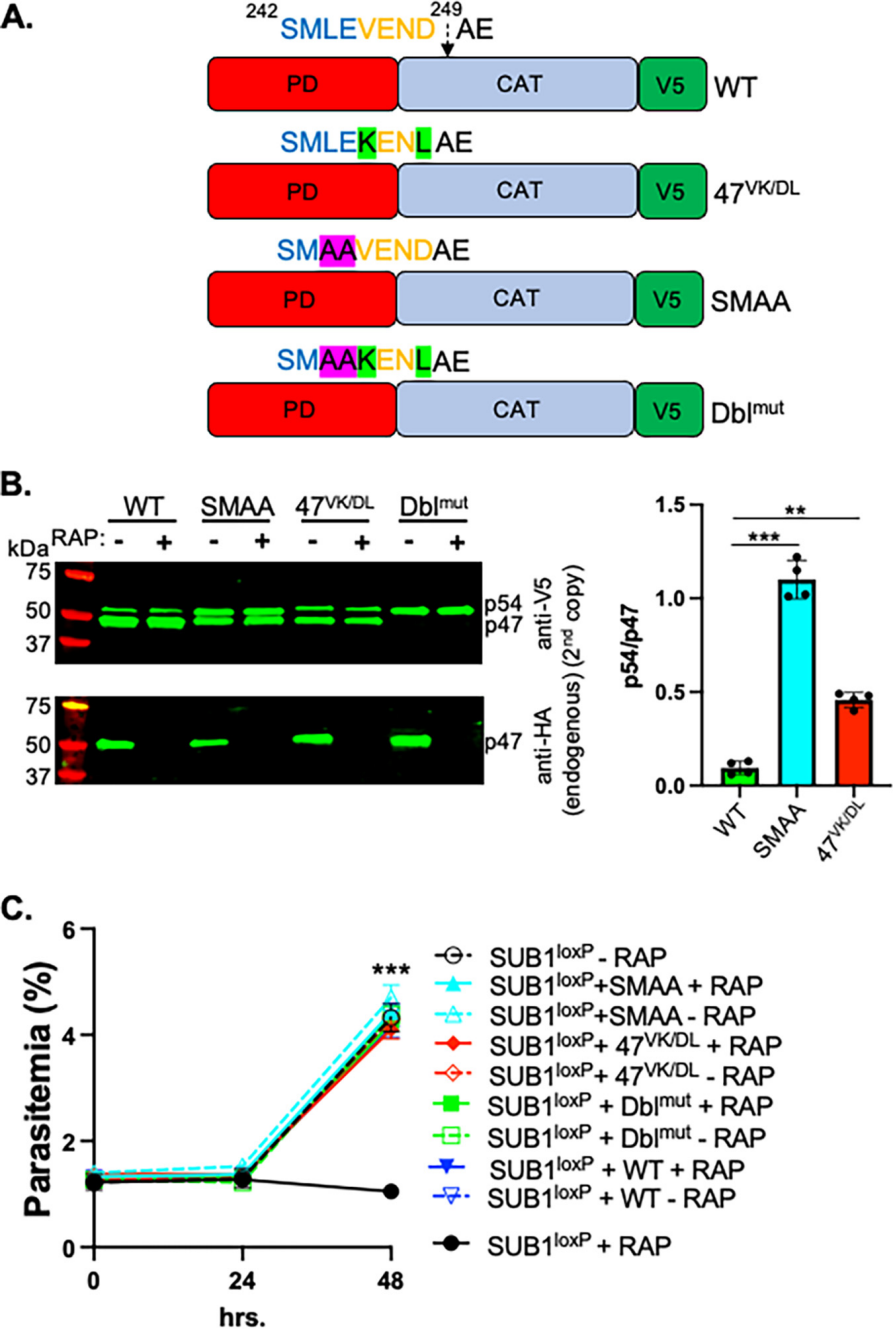
Conversion of p54 to p47 can be autocatalytic or PM X-mediated and is dispensable for parasite growth. (A) Schematics of the wild type (WT) or the different cleavage mutant SUB1 constructs that were tagged at the C terminus with V5 epitope and expressed as second copies in the SUB1^loxP^ transgenic line. (B, Left) Western blotting showing the processing of the WT or the different cleavage mutants SUB1 expressed as second copies (top, V5) in the presence (−RAP) or absence (+RAP) of the endogenous SUB1 (bottom, HA). Samples were harvested from synchronized 44- to 47-h-old schizonts that were treated with 10 nM rapamycin (RAP) in the ring stage for 3 h. (B, Right) Quantification from three independent Western blots as on the left. Error bars represent standard deviation. (C) Synchronized parasites were cultured as in panel B. However, the initial parasitemia was set to 1%. Mean values from three independent experiments are shown, and error bars represent standard deviations. Statistical two-tailed Student's *t* test was done to determine the significance of difference in growth between the parasites lacking endogenous SUB1 (SUB1^loxP^ + RAP) and parasites expressing only double-cleavage mutants as in Fig. 4B (Dbl^mut^ + RAP). **, *P < *0.01; ***, *P < *0.001.

An earlier study reported that the p54-to-p47 conversion for rSUB1 can occur through autocatalytic cleavage after the aspartate within the ^242^SMLEVENDAE^251^ motif (11). However, our *in vitro* cleavage assay, as depicted in Fig. 3, suggested that PM X could cleave both p31 and the p54 of SUB1. Consistent with this, previously, a recombinant form of PM X has been shown to cleave the ^242^SMLEVENDAE^251^ peptide after the methionine (14). Therefore, to elucidate the processing of the p54 to p47 further, we moved to cultured parasites and expressed either WT or cleavage site mutant forms of SUB1 as C-terminally V5-tagged second copies (Fig. 4A). For the autocatalytic cleavage mutant, we mutated the V^246^ to K and the D^249^ to L together in one construct (47^VK/DL^). This dimutation was previously shown to abolish cleavage of a peptide substrate by rSUB1 (22). As a PM X cleavage mutant, we mutated the LE to AA in the ^242^SMLE^245^ sequence (^242^SMAA) (14). Finally, in one construct, we mutated both the p47 autocleavage and the proposed PM X cleavage sites together (Dbl^mut^). Besides looking at the processing of the different cleavage mutants, we wanted to determine if they were functional or not. For this, we expressed the second-copy mutants in an endogenous SUB1-inducible knockout line, SUB1^loxP^. In this line, the parasites stably express the rapamycin (RAP)-inducible dimerizable Cre recombinase (DiCre) (23). To excise the segment of the SUB1 gene encoding the catalytic residues upon RAP treatment, we replaced the *sub1* locus with a human-recodonized version of *sub1* carrying loxP sequences and simultaneously added 3×HA at the C-terminal end as described earlier (9, 24) (Fig. S3A). PCR (Fig. S3B, left) and Western blotting (Fig. S2B, right) demonstrated rapid and efficient RAP-induced excision of the floxed DNA sequences and ablation of SUB1 expression. As shown earlier (9), addition of RAP to synchronized ring-stage cultures of the SUB1^loxP^ parasites led to a severe growth defect that was detectable from the second cycle on.

10.1128/mbio.00673-23.3 FIG S3 Generation of the rapamycin (RAP)-inducible SUB1 knockout (KO) parasite line. (A) Schematic of the linear donor vector used to replace the endogenous locus with an HA-tagged human recodonized *sub1* (human R.C. *sub1*) by CRISPR/Cas9-mediated genome editing. Also shown is the modified locus before and after treatment with RAP. Positions of the forward (278) and the reverse (279) primers used to check genome editing by PCR are shown. (B, Left) PCR. (B, Right) Western blotting showing excision of a segment of the SUB1 ORF and the resulting loss of protein expression (HA), respectively. As a loading control for the Western blotting, expression of the ER-resident protease plasmepsin V (PM V) was used. (C) Replication of mock (DMSO)-treated (−RAP) or RAP-treated (+RAP) parasites over a period of 96 h. The experiment was performed two times. Shown are the mean values; error bars represent standard deviation. Download FIG S3, TIF file, 2.0 MB. Copyright © 2023 Mukherjee et al. 2023 Mukherjee et al. https://creativecommons.org/licenses/by/4.0/ This content is distributed under the terms of the Creative Commons Attribution 4.0 International license.

To understand cleavage of the second-copy SUB1, we analyzed the protein expression by Western blotting in synchronized schizonts (44 to 47 h old) that were either pretreated with RAP or not. Aliquots of the same samples were cultured further and tested for rescue of the growth defect due to depletion of endogenous SUB1. Under conditions when the endogenous SUB1 was processed to p47, the WT second copy was detected primarily as the p47 as well (Fig. 4B). However, both the 47^VK/DL^ and the ^242^SMAA mutants demonstrated inefficient conversion of p54 to p47. Moreover, the degree of the defect was more pronounced for the ^242^SMAA mutant than the 47^VK/DL^. Nevertheless, the fact that both were able to partially process to p47 suggested that the two sites could be alternatively used. Consistent with this, the Dbl^mut^ was detected only as p54, suggesting a complete blockage in the p54-to-p47 conversion. Importantly, each of the cleavage site mutants, including the Dbl^mut^, was able to fully rescue growth in the absence of endogenous SUB1 (Fig. 4C). This suggested that the p54-to-p47 conversion is dispensable for SUB1 functions in the parasites.

### Primary processing of SUB1 is strictly autocatalytic and is required for intracellular trafficking of SUB1.

**FIG 5:**
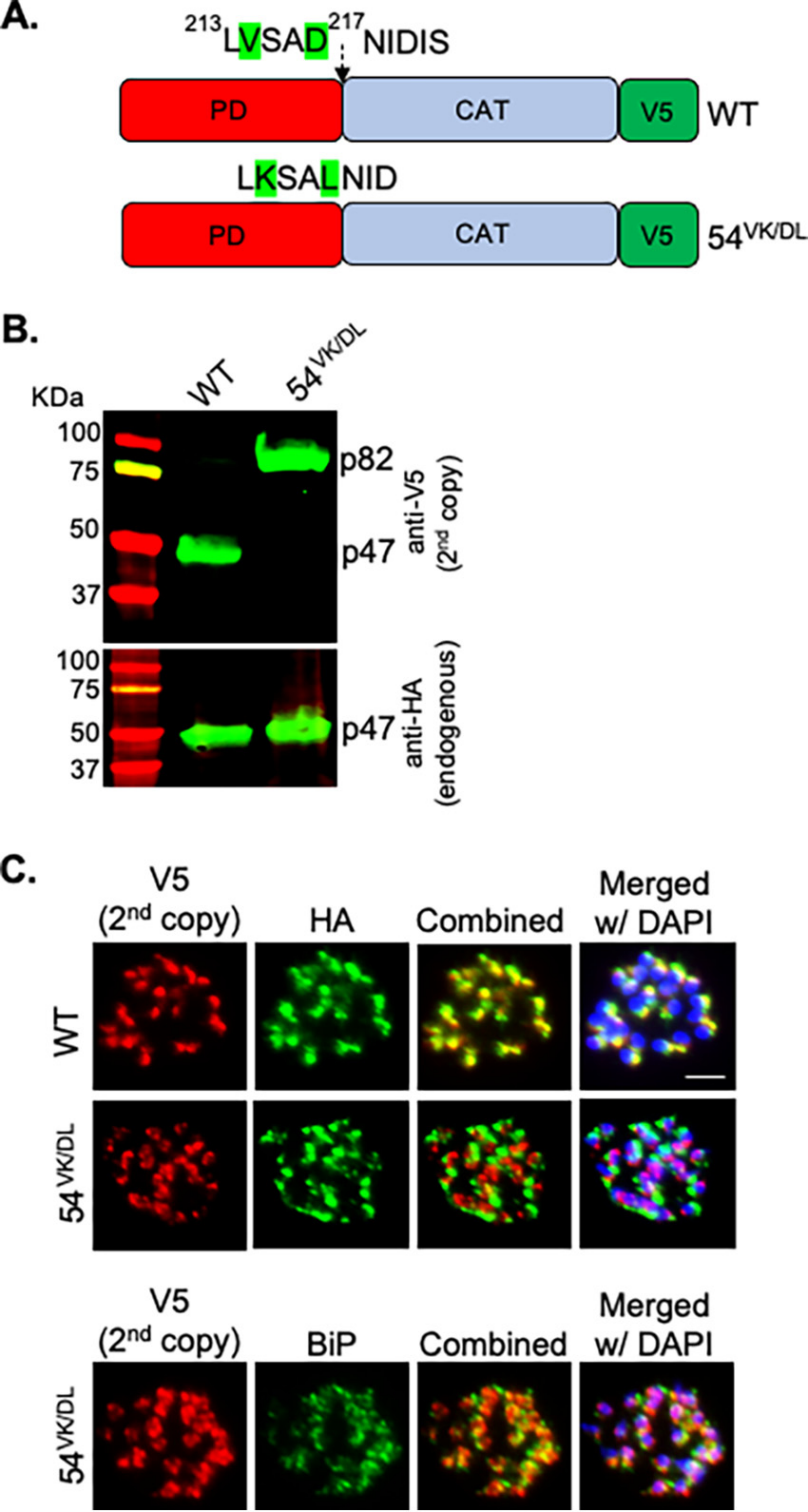
Primary processing of the SUB1 precursor to p54 is autocatalytic and is required for intracellular trafficking. (A) Schematics of the wild-type (WT) or the p54 cleavage mutant (54^VK/DL^) constructs that were tagged at the C terminus with V5 epitope and expressed as the second copy in the SUB1^loxP^ transgenic line. (B) Western blotting showing the processing of the WT or the 54^VK/DL^ mutant SUB1 expressed as second copies (top, V5) and the endogenous SUB1 (bottom, HA). Samples were harvested from synchronized 44- to 47-h-old schizonts. The experiment was repeated three times. Shown is a representative blot. (C) Representative images from IFAs showing that the WT second copy (V5), but not the 54^VK/DL^ mutant (V5), was colocalized with the endogenous SUB1 (HA). The 54^VK/DL^ mutant, however, was colocalized with the ER marker BiP. Scale bar, 2 μm. Experiments were repeated two times.

Analysis of the p47 cleavage mutant forms of SUB1, as described in the previous section, suggested two alternative pathways for p54-to-p47 conversion, one PM X mediated and another by autocatalysis. To test the same for the primary processing of SUB1 from p82 to p54, we carried out similar experiments as in the previous section, but this time, we mutated the V^214^ to K and the D^217^ to L within the proposed p54 autocleavage site (^213^LVSAD NID^220^, arrow indicates the scissile bond) (Fig. 5A, 54^VK/DL^) (11). While both the endogenous SUB1 and the WT second copy were processed to p47, the 54^VK/DL^ mutant accumulated in the precursor p82 form (Fig. 5B). Additionally, immunofluorescence assay (IFA) microscopy with mature schizonts showed that the WT second-copy SUB1, but not the 54^VK/DL^ mutant, was colocalized with the endogenous SUB1 in the exonemes (Fig. 5C, top and middle). The 54^VK/DL^ mutant, on the other hand, showed near-complete colocalization with the ER marker BiP (Fig. 5C, bottom). Finally, consistent with the inhibitory function of p31, the parasite growth assay revealed that the 54^VK/DL^ mutant failed to rescue the growth defect due to the depletion of the endogenous SUB1 (Fig. S4). Together, these data suggested that, unlike the secondary processing, the primary conversion of SUB1 precursor to p54 is strictly autocatalytic and is required for SUB1’s intracellular trafficking.

10.1128/mbio.00673-23.4 FIG S4 Primary autocatalytic processing of SUB1 is essential for parasite growth. Synchronized parasites from the indicated lines were cultured were cultured as in Fig. 5. Mean values from three independent experiments are shown, and error bars represent standard deviations. A statistical two-tailed Student’s *t*-test was done to determine the significance of the difference in growth between the 54^VK/DL^ mutant parasites grown in the presence and absence of RAP. ***, *P < *0.001. Download FIG S4, TIF file, 2.0 MB. Copyright © 2023 Mukherjee et al. 2023 Mukherjee et al. https://creativecommons.org/licenses/by/4.0/ This content is distributed under the terms of the Creative Commons Attribution 4.0 International license.

## DISCUSSION

Egress of malaria parasites from infected host RBCs is an essential process for propagation. The serine protease SUB1 plays a central role in this process by proteolytically activating several downstream effectors that mediate the breakdown of the PVM and the RBCM. SUB1, like other subtilisins, is synthesized as a proenzyme (p82) with an N-terminal prodomain (PD). The PD facilitates the folding of the catalytic domain of SUB1 while acting as a tightly bound inhibitor for the cognate enzyme (19). The existing model for proenzyme processing based on a recombinant SUB1 (rSUB1) suggests that both the primary processing of p82 to the p31-p54 intermediate complex and the subsequent conversion into p47 are dependent on the autocatalytic activity of SUB1 (11). We and others have shown that the conversion of p54 to p47 in parasites is dependent on the upstream protease PM X (12–14). Importantly, depletion of PM X in parasites renders SUB1 nonfunctional, suggesting a crucial role for PM X in the activation of SUB1. In this current study, we investigated the mechanism by which PM X regulates SUB1 activity. We demonstrated that in the absence of PM X, SUB1 accumulates as the p31-p54 complex in P. falciparum (Fig. 1). *In vitro*, we showed that both p31 and p54 are direct substrates of PM X (Fig. 2). The mutant p54 that did not get processed to p47 was fully functional inside the parasites (Fig. 4C). Taken together, these data suggest that PM X-mediated cleavage of p31 in the p31-p54 complex is the crucial step for SUB1 activation. The present study, therefore, links the loss of SUB1 activity in the absence of PM X to the maintenance of p31 attached to p54.

**FIG 6:**
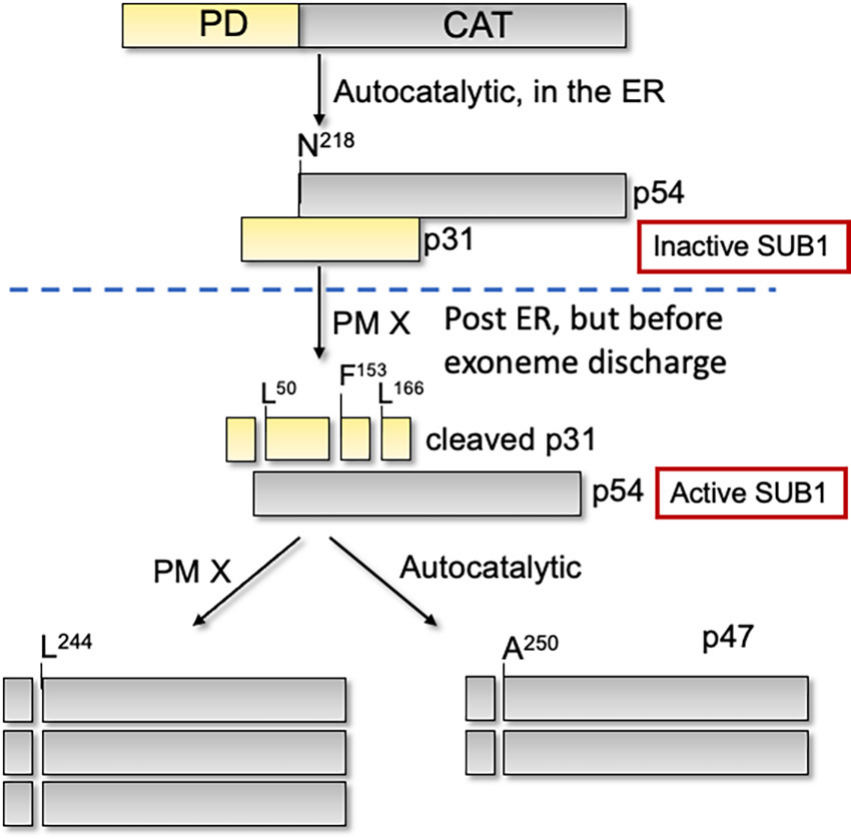
Model for the activation of SUB1 in Plasmodium falciparum as suggested by the present data.

Using the E. coli-expressed p31, we showed that PM X can cleave p31 at three sites, one toward the N terminus (^48^RFLE^51^) and the other two close to the C terminus (^151^SFFQ^154^ and ^164^SFLE^167^) (Fig. 2). Of the two C-terminal sites, cleavage at ^164^SFLE^167^ was detected in WT p31. However, cleavage at the ^151^SFFQ^154^ site was detected only when the ^164^SFLE^167^ sequence was mutated. One possibility is that ^164^SFLE^167^ is the preferred sequence for PM X in WT p31. Alternatively, both the ^164^SFLE^167^ and the ^151^SFFQ^154^ sequences could be targeted by PM X on the same protein. These two cleavage sequences are only 13 amino acids apart from each other. Therefore, it is possible that the small polypeptide generated due to cleavage at these two sites ran off the SDS-PAGE, preventing us from detecting it. Importantly, all three cleavage sequences on p31 are conserved in SUB1 from multiple species of *Plasmodium* (21). The ^48^RFLE^51^ sequence is part of an alpha helix that has been shown to fold over and interact with the active site in the crystal structure of *Pv*SUB1 (21). The C-terminal cleavage sequences (^151^SFFQ^154^ and ^164^SFLE^167^), on the other hand, belong to a segment of p31 which folds into a structure similar to the proregion from bacterial subtilisins and mediates the inhibition of the protease activity (20, 21). One consequence of p31 cleavage, therefore, could be the disruption of the interaction between p31 and p54. Second, it could lead to a change in the conformation of the inhibitory residues of p31 lodged in the active site. In fact, truncation at the C-terminal end of p31 has been shown to substantially reduce the inhibition of SUB1 activity *in vitro* (19). Whether, inside the parasites, PM X targets the *in vitro*-identified cleavage sequences on p31 remains unknown. Mutations at these sequences in the parasites led to the accumulation of the mutant SUB1 in the precursor form (p82) that remained sequestered in the parasite ER, possibly due to the misfolding of the protease (data not shown). Nevertheless, the accumulation of full-length p31 in parasites only in the absence of PM X, together with the findings from the *in vitro* assays, evokes a model in which SUB1 maturation involves direct cleavage of p31 by PM X to destabilize the inhibitory p31-p54 complex (Fig. 6). It is worth noting that there was a small amount of a 27-kDa band that came down along with the catalytic domain after *in vivo* processing by PM X (Fig. 1B). This is likely to be a partially cleaved p31. Its size is similar to that of RFAA mutant p31 and therefore may represent a cleavage at the C terminus.

Another important finding that comes from our study is that the conversion of the p54 to p47 is dispensable for parasite growth. Previously, p47 was assumed to be the active form of SUB1. Nevertheless, since the recombinantly made p54 was purified as a complex with p31, it was not possible to determine the activity of the p54. Moreover, our attempt to purify p54 without a PD also failed. Therefore, we took advantage of the previously published data to mutate the potential p47 autocleavage site and determine the fate of the mutant SUB1 directly in the parasites (11). This revealed that the conversion of p54 to the terminal p47 form can be effected by either a direct cleavage by PM X or by less efficient autocatalysis. Currently, the cellular compartment where the second processing of SUB1 takes place remains unknown. However, in theory, this must take place in a space that maintains enough acidity for PM X to be functional. Although the effect of pH in controlling the SUB1 activity has not been tested, a redox-sensitive, solvent-exposed disulfide bond has been shown to be critical for SUB1 activity (20). An acidic environment (potentially in the exoneme) could, therefore, maintain the processed SUB1 in an inactive state.

Unlike the p54-to-p47 conversion, the primary processing of SUB1 in the parasite is strictly autocatalytic in nature (Fig. 6B). Importantly, a mutant SUB1 that failed to cleave itself at the PD-p54 junction was retained in the parasite ER. This suggested that like other subtilisins (25), the primary processing of SUB1 is critical for intracellular trafficking of the protease. Whether trafficking through the Golgi requires distinct SUB1-folding intermediates remains to be tested.

Subtilisins, in general, are synthesized as inactive proenzymes that are activated upon reaching the site of action (25–29). Typically, the PD facilitates folding of the rest of the proenzyme. Besides acting as a chaperone, the PD also acts as an endogenous inhibitor of the protease activity. When activation is required, there is a release and/or autocatalytic degradation of the PD that can happen both in *cis* or in *trans*. For most subtilisins, the PD displacement has been proposed to be the rate-limiting step and occurs due to pH changes during translocation to the site of activity. Some of the PDs have been proposed to have pH sensors that trigger the process (30). *Pf*SUB1 appears to use a novel strategy for activation. It requires an endogenous activating protease, PM X, that, by destroying the propiece, relieves inhibition. Why the malaria parasite does it this way is unclear. Perhaps the environment of the exoneme is not conducive to the conformational change needed for automaturation.

## MATERIALS AND METHODS

### Reagents.

All primers were obtained from Integrated DNA Technologies. A list of primers used in this study can be found in Table S1 in the supplemental material. Restriction enzymes were purchased from New England Biolabs. HisTrap magnetic Sepharose beads were from Cytiva. The Expi293 cells, transfection reagent ExpiFectamine, blasticidin S HCl, Ni-nitrilotriacetic acid (NTA) beads, mouse anti-HA antibody-coated magnetic beads, and rabbit anti-V5 antibody were from Thermo Fisher. The rat anti-FLAG antibody was from Novus Biologicals; the rabbit anti-HA antibody, the rabbit anti-His antibody, and rapamycin were from Sigma. The mouse anti-PM V antibody was previously described (31). Anhydrotetracycline (aTc) was purchased from Cayman Chemical. DSM-1 was from Asinex.

10.1128/mbio.00673-23.5 TABLE S1 List of primers used in this study. Download Table S1, TIF file, 2.0 MB. Copyright © 2023 Mukherjee et al. 2023 Mukherjee et al. https://creativecommons.org/licenses/by/4.0/ This content is distributed under the terms of the Creative Commons Attribution 4.0 International license.

### Generation of plasmids.

To epitope tag the N terminus of the SUB1 p31 at the endogenous locus in the previously reported transgenic PM X^apt^ parasites (12), a human recodonized gene block was obtained from IDT. This gene block had the following elements in the order: signal peptide of SUB1-3×HA-remaining open reading frame (ORF) of SUB1. To generate the donor vector for the CRISPR/Cas9-mediated genome editing, 440 bp of the 5′ untranslated region (5′ UTR) immediately upstream of the start *sub1* (left homologous region [LHR]) and 440 bp of the 3′ UTR (right homologous region [RHR]) were PCR amplified from the genomic DNA of the NF54 strain of P. falciparum using the primer pairs 272-273 and 270-271, respectively. The SUB1 gene block was PCR amplified using primer pair 274-275. An assembly PCR was performed using the primer pair 272-275 and a mixture of LHR, RHR, and SUB1 gene blocks as the template. The amplified fragment was subsequently cloned into the plasmid PM2GT (32) to generate the plasmid PM2GT-HA-p31-SUB1. To generate the SUB1 conditional knockout parasite line, a yPM2GT-SUB1^loxP^ plasmid was used following the principle described earlier (9). The yPM2GT vector has been reported earlier (12). First, the 5′ and 3′ homology arms were PCR amplified from NF54 gDNA using primer pairs 259-261 and 258-260, respectively. A Sera2 intron containing a loxP site in the middle (9) was obtained as a gene block and further PCR amplified using the primer pair 262-264. A recodonized fragment of the SUB1 ORF was obtained as a gene block and further PCR amplified using the primer pair 263-265. Finally, an assembly PCR was performed using the primer pair 259-265 and a mixture of 5′ and 3′ homology arms, Sera2 intron, and the recodonized fragment of SUB1 as the template. The amplified fragment was cloned into the yPM2GT plasmid that was digested with XhoI and NheI. A second Sera2 intron with loxP site was amplified using the primer pair 276-277 and cloned into the above-described plasmid that was digested with SalI and EcoRI. This resulted in the final yPM2GT-SUB1^loxP^ plasmid. This plasmid was linearized with AflII and used as the donor vector for CRISPR/Cas9-based genome editing.

To complement the SUB1 knockout, we modified the previously described plasmid pEOE-pAMA1-2× attP^13^. To make the expression of the second-copy SUB1 stage specific, we used a 2,000-bp sequence located upstream of the PM X coding region (pPM X). This putative promoter-containing sequence was PCR amplified from the NF54 genomic DNA using the primer pair 278-279 and ligated between the AflII and XhoI restriction enzymes cut sites within pEOE-pAMA1-2× attP to generate the pEOE-pPM X-2X attP. The ORF of SUB1 was amplified using the primers 280 and 281. The amplified fragment was subsequently fused by Gibson assembly with the pEOE-pPM X-2X attP that was previously digested with XhoI and EagI. This resulted in the production of the final WT complementation construct pEOE-SUB1-V5-2XattP. Subsequent site-directed mutagenesis (QuikChange Lightning; Agilent) on this plasmid used primers 266, 267, 268, and 269 to generate the different SUB1 cleavage mutant constructs.

To generate the bacteria expression plasmid to produce rp31 from E. coli, the pET28a plasmid (Novagen) was used. First, the 3×HA sequence was PCR amplified from the yPM2GT-SUB1^loxP^ plasmid using the primer pair 280-281 and subsequently cloned between the NcoI and XhoI sites in the pET28a plasmid. A bacteria-recodonized version of the p31 sequence of SUB1 was then cloned into the modified pET28a at the XhoI site, thus labeling the N and the C termini of rp31 with 3×HA and 6×His, respectively. The resultant pET28a-SUB1-p31 plasmid was used as a template to make the different rp31 cleavage site mutants by site-directed mutagenesis using the primers 254, 255, and 256.

Generation of the pHLSec-SUB1 plasmid to express SUB1 from the mammalian cells has been described earlier (12). To epitope tag the N terminus of p31, the 3×HA sequence was PCR amplified from the yPM2GT-SUB1^loxP^ plasmid using the primer pair 284-285 and cloned into the pHLSec-SUB1 plasmid at the AflIII site.

### Parasite culture, transfection, and synchronization.

NF54attB parasites with stably integrated Cre recombinase cassette (DiCre) (23) and the resultant transgenic strains were cultured in human red blood cells using RPMI with 0.1% Albumax, as previously described (33). For the SUB1^loxP^ line, following electroporation, the transfectants were selected in the presence of 2 μM DSM-1 (34). For complementation of SUB1, plasmids carrying WT or mutant SUB1 coding sequences were independently cotransfected with a Bxb1 integrase plasmid (35) into the SUB1^loxP^ parasites. Transfectants were selected with 5 nM WR99210 (34), together with DSM-1. PM X^apt^ parasites (12) were maintained in the presence of 100 nM aTc and 5 μg/mL blasticidin S (34). For tagging the N terminus of the endogenous SUB1, the PM X^apt^ parasites were cotransfected with the linearized plasmid PM2GT-HA-p31-SUB1 and the guide DNA plasmid. Transfected parasites were selected with 5 nM WR99210, together with 2.5 μg/mL blasticidin and 100 nM aTc. Integration of all plasmids was verified by PCR. For CRISPR/Cas9-mediated editing of the *sub1* locus, we used the guide sequence GCATTAACTAGTACATCAAA.

Highly synchronous ring-stage parasites were obtained as follows. High-parasitemia schizont cultures were passed through marker-assisted congenic screening (MACS) LD magnet columns (Miltenyi Biotec), and schizonts were collected. These were then added to fresh uninfected RBCs resuspended in warm culture media. The cultures were shaken at 80 rpm for 3 h, and the resulting parasites were synchronized using 5% sorbitol as described before (36). To induce SUB1 knockout, synchronized ring-stage parasites (0 to 3 h old) with floxed *sub1* locus were treated with 10 nM rapamycin for 3 h. Parasites were then washed in prewarmed media and cultured to mature schizont stage (44 to 47 h postinvasion). Genomic DNA was extracted, and the excision of the floxed segment of SUB1 ORF was analyzed by PCR using the primer pair 282-283.

### Parasite growth assay.

For growth curves, synchronized ring-stage parasites were diluted to an initial 1% parasitemia at 2% hematocrit. Cultures were split, and rapamycin (10 nM) was added to appropriate wells. Control wells received equal volumes of dimethyl sulfoxide (DMSO; vehicle). Parasitemia was determined every 24 h using flow cytometry as described previously (12).

### Expression and purification of recombinant SUB1 from mammalian cells.

rSUB1 was purified as a secreted protease from the mammalian cells using the protocol described before (22) with some minor changes. Briefly, the Expi-HEK293 cells (Thermo Scientific) were transfected with the mammalian expression plasmid pHLSec containing the recodonized SUB1. For DNA delivery, ExpiFectamine was used as per the manufacturer’s recommendation. Tunicamycin was added at a final concentration of 0.5 μg/mL immediately after transfection, and the cultures were grown for 72 h. Culture supernatants were then passed through a 0.22-μm filter and diluted with 3 culture volumes of 50 mM Tris-Cl, pH 8.0, 100 mM NaCl, and 10 mM imidazole. The diluted supernatant was purified using the Ni-NTA resin (Thermo Fisher). After extensive wash in the same buffer, the protein was eluted with 50 mM Tris-Cl, pH 8.0, 100 mM NaCl, and 400 mM imidazole. The eluted sample was then buffer exchanged using an Amicon Ultra 3-kDa-molecular-weight cutoff filter (Millipore, USA) and finally resuspended in 20 mM Tris, pH 7.6, supplemented with glycerol to 10% (wt/vol), and stored in aliquots at −70°C.

### Expression and purification of recombinant p31 from E. coli.

For rp31, the E. coli expression system was used. Briefly, the BL21(DE3) competent cells were transformed with the pET28a plasmid containing the SUB1-p31 with 3×HA and 6×His as the N- and C-terminal tags, respectively. Ten milliliters of bacterial culture was grown overnight at 37°C and diluted to a 500-mL culture with a starting optical density at 600 nm (OD_600_) of 0.01. When the OD_600_ reached ~0.6, 250 μM IPTG (isopropyl-β-D-thiogalactopyranoside) was added, and the cultures were grown overnight at 18°C. The cultures were then pelleted and lysed in 0.2 g lysozyme, 100 mM Tris, pH 8.0, with 0.1% Triton X-100 by sonication. The lysed bacteria were spun down at 12,000 rpm for 10 min. The lysate was diluted 20× in 100 mM NaCl, 50 mM Tris, pH 8.0, and 10 mM imidazole and passed over the Ni-NTA column. The column was washed with 10 column volumes of buffer containing 100 mM NaCl, 50 mM Tris, pH 8.0, and 25 mM imidazole. The bound proteins were then eluted with 100 mM NaCl, 50 mM Tris, pH 8.0, and 500 mM imidazole. Finally, the proteins were buffer exchanged (100 mM NaCl, 50 mM Tris, pH 7.5) and concentrated with an Amicon Ultra 3-kDa-molecular-weight cutoff filter (Millipore, USA). The concentrated proteins were then immediately used for *in vitro* rPM X cleavage assay.

### *In vitro* rPM X cleavage assay.

Activity of rPM X toward rp31 was carried out as previously described (13) with minor modifications. Briefly, 250 ng of rPM X was incubated with 1 μg of rp31 in 40 μL of PM X activity buffer (150 mM NaCl, 25 mM MES [morpholineethanesulfonic acid], and 25 mM Tris, pH 5.5) for 3 h at 37°C. To inhibit PM X activity, 1 μM CW-117 (12, 37) was added to the samples at pH 5.5. Following incubation, samples were mixed with 4× sample loading buffer and heated at 99°C for 5 min. Fractions of equal volumes were subjected to SDS-PAGE and immunoblotting.

For cleavage of rSUB1 by rPM X, at first, the former was immobilized onto HisTrap magnetic Sepharose beads as per the manufacturer’s protocol. Protein-bound beads were washed 2× in 150 mM NaCl and 50 mM Tris, pH 7.6, and subsequently resuspended in PM X activity buffer. rPM X and CW-117 were added to the appropriate tubes, and reaction mixtures were incubated as described above. Following incubation, the tubes were placed on magnets, and the supernatants were separated. The bead-bound proteins were eluted with buffer containing 300 mM NaCl, 50 mM Tris, and 500 mM imidazole, pH 8.0. Both the supernatant and the eluted fractions were processed as described above.

### Immunoprecipitation and Western blot analysis.

To immunoprecipitate p31 from parasite lysates, highly synchronized PM X^apt^ parasites expressing N-terminally 3×HA-tagged SUB1 were split into two plates. To one plate, aTc was added, and to the other, an equal volume of vehicle (DMSO) was added. Forty-six hours postinvasion, free schizonts were collected by saponin permeabilization. Samples were subsequently lysed in 150 mM NaCl, 50 mM Tris, pH 7.5, and 1% NP-40 supplemented with protease inhibitor cocktail. Insoluble material was removed by centrifugation. Clarified lysates were mixed with the Dynabeads coated with mouse anti-HA antibody. Immunoprecipitation was carried out as per the manufacturer’s recommendation. Both the immunoprecipitated and the flowthrough fractions were mixed with 4× sample loading buffer and heated at 99°C for 5 min. Aliquots of equal cell equivalents were subjected to SDS-PAGE and immunoblotting.

For SUB1 knockout Western blotting, following synchronization, the SUB1^loxP^ parasites expressing a second copy of SUB1 or not were cultured in the presence or absence of rapamycin as in the parasite growth assay, except the parasitemia was maintained around 5%. Forty-six hours postinvasion, samples were harvested as described above and lysed with radioimmunoprecipitation assay (RIPA) buffer, and insoluble fractions were removed by centrifugation. Cell lysates were then directly boiled with SDS-PAGE loading buffer followed by Western blotting.

Primary antibodies included rabbit anti-HA (1:1,000), rabbit anti-SUB1 (1:1,000), rabbit anti-HA (1:1,000), rabbit anti-V5 (1:1,000), mouse anti-PM V (1:500), mouse anti-His (1:1,000), and rat anti-FLAG (1:1,000). For all, appropriate IRDye-conjugated secondary antibodies were used at 1:10,000 dilution. Blots were visualized on an Odyssey imaging system (Li-Cor).

### Immunofluorescence assays.

IFAs were performed as before (13). Briefly, synchronized 44- to 47-h-old schizont cultures were smeared on glass slides, fixed in 3.7% paraformaldehyde for 15 min, and blocked in 3% bovine serum albumin (BSA) in phosphate-buffered saline (PBS) overnight at 4°C before antibody staining. The antibodies used for IFA were rabbit anti-HA (1:500), mouse anti-V5 (1:500), and mouse anti-BiP (1:500). The secondary antibodies were used as 1:2,000 dilutions and were conjugated to Alexa Fluor 488 or 546 (Life Technologies). Cells were mounted with ProLong and 4′,6-diamidino-2-phenylindole (DAPI) (Invitrogen) and imaged using a Zeiss Imager M2 Plus widefield fluorescence microscope, using a 63× objective and the AxioVision 4.8 software for epifluorescence imaging.

### Statistical analysis.

Unless specified otherwise, all values in all figures are averaged from three independent biological repeats, and error bars represent standard deviations. Differences were assessed by two-tailed Student's *t* test using Microsoft Excel software. *P* values indicating statistical significance were grouped in all figures. All graphs were prepared using GraphPad Prism 8.
