# Supplementary material for: Activation of the Plasmodium Egress Effector Subtilisin-Like Protease 1 Is Mediated by Plasmepsin X Destruction of the Prodomain
Source: mBio. 2023 Apr 10;14(2):e00673-23. doi: 10.1128/mbio.00673-23 (PMC10128010; doi:10.1128/mbio.00673-23)
Supplement: DATA SET S1 [file mbio.00673-23-s0006.pdf]

Iowa State University
Protein Facility
PROTEIN/PEPTIDE SEQUENCE REPORT

Date: August 8, 2022

To: Sumit Mukherjee

Sample Number: 10022

Sample Name: 2

Sample Preparation: The membrane was washed with DI water and loaded onto the instrument for sequence analysis.

Instrument: Shimadzu PPSQ-53A

Sequencing Method: Edman Degradation

Cycle Number	Amino Acid
1	M
2	R
3	E
4	L

The major amino acid is listed first for each cycle. If you have any questions, feel free to contact me.

Prepared by: Joel Nott
Tel: 515-294-3267, protein@iastate.edu

Protein/Peptide Sequencing Submission Form

Tracking Number-1550: 10022 Date: Aug-03-2022

Your sample ID 2

Name: SUMIT MUKHERJEE	Login: sumitmukh	Principal Investigator: Daniel Goldberg
Department/Company: Washington University School of Medicine	Phone #: 8062245077	Fax #:
E-Mail Address: sumit.mukherjee@wustl.edu	Principal Investigator E-Mail Address: dgoldberg@wustl.edu	
Mailing Address: 660 South Euclid Avenue, Department of Molecular Microbiology, Saint Louis, Missouri, 63110		
Account #: PR00136711		Assignee:
Business Purpose:		
Billing Contact Name: Rachel Warhover		
Billing Mailing Address: 4990 Children's Place, Infectious Diseases Division, Saint Louis, Missouri, 63110		
Billing Phone #: 314-454-8225	Billing E-Mail Address: rachelwarhover@wustl.edu	

✓ I agree to the terms and conditions present at http://www.biotech.iastate.edu/facilities/Agreements/ProteinTechnicalServicesAgreement.pdf

How many residues do you need? 4

Sample Information

Sample amount________moles;or ________microgramsM.W.________

For samples in solution: What solvent is the sample in?________

For samples electroblotted toPVDF: What membrane was used?

____Immobilon-P (.45 micron) (Millipore) ____Problot (.1 micron) (ABI)
____Westran (.45 micron) (Schleicher & Schuell) ____Trans-Blot (.1 micron) (Biorad)
____Immobilon-PSQ (.1 micron) (Millipore) ____Fluoratrans(.1 micron) (Pall Corp)

N-Terminal blocked: No________Do not know________ Yes________

Protein/PeptideModified: Yes, at________ with________

Cysteine modified: Yes________ If yes, what derivative?________ No________

Enzyme treatment: Yes________ What enzyme?________ Cleavage sites________

Radioactivity: Yes________ No________

Protein sequence known: Yes______ No______ DNA sequence known: Yes______No______

Describe purification stepsin detail, especially possible contaminants such as buffer, salts, and SDS:

If your sample was collectedon an HPLC, please attachthe chromatogram with AUFS, gradient, solvents, column and wavelength.

[Sequence Analysis]

Data Acquired	:	8/7/2022 12:32:56 PM
Data Processed	:	8/8/2022 7:25:40 AM
Reactor	:	2
Number of Cycles	:	5
Sequence Schedule	:	C:\PPSQ\SeqProg3_PDA_BGE\PVDF9-3G.sch
Sample Name	:	Sumit Mukherjee, 2
Sample Amount(pmol)	:	10.0
Sample ID	:	10022
Operator Name	:	System Administrator
Data File	:	10022_08-07-2022
Start Number	:	1
Method File	:	10022_08-07-2022.lcm
Batch File	:	10022_08-07-2022.lcb
Data Folder Path	:	C:\LabSolutions\Data\Project1\PPSQ\10022_08-07-2022
Number of Analyses	:	5 / 5
Standard File	:	C:\LabSolutions\Data\Project1\PPSQ\10022_08-07-2022\PTH-AA_08-04-2022_D01.lcd
Data Comment		

[Sequence]

M R E L

[Estimated Sequence]

	1	2	3	4
1st	M	R	E	L
2nd	V	I	Y	K
3rd	F	Q	G	N
4th	S	D	K	W
Reliability(%)	100.0	33.8	59.5	91.6

[Evaluated Value]

	1	2	3	4
D	0.45	131.06	0.63	0.81
E	0.29	96.45	4279.12	0.53
N	0.64	55.40	106.56	72.07
S	2.10	0.87	106.63	0.93
T	0.67	73.33	76.97	0.78
Q	0.38	131.38	0.58	0.87
G	0.68	63.75	235.22	0.73
H	0.77	1.00	4.00	0.76
A	0.67	0.99	0.72	0.90
R	0.09	1196.95	0.52	0.34
Y	0.79	18.13	765.12	0.31
P	0.35	18.99	85.04	0.32
M	3777.23	0.42	0.07	0.16
V	378.38	0.45	0.39	1.00

W	0.49	0.83	0.89	2.16
K	0.33	74.37	115.06	622.33
F	52.96	0.37	54.74	0.41
I	0.07	354.46	0.46	0.09
L	0.76	82.85	0.53	5254.12

[Amount Yield(pmol)]

	1	2	3	4
D	5.77	6.62	1.63	0.00
E	1.59	3.61	123.63	0.00
N	1.75	1.36	2.08	90.04
S	3.38	0.00	6.65	74.77
T	2.76	5.11	3.43	0.00
Q	1.56	6.19	0.00	0.00
G	4.16	6.68	7.13	0.00
H	0.88	12.58	5.79	15.72
A	3.98	15.41	24.37	42.52
R	4.35	26.27	16.89	0.00
Y	3.14	12.09	48.31	0.00
P	0.00	0.00	78.66	0.00
M	90.65	0.00	27.28	0.00
V	8.40	54.36	99.50	17.19
W	2.91	32.67	11.57	4.13
K	6.55	65.63	22.15	17.56
F	0.00	83.11	25.35	8.19
I	2.90	68.56	0.00	0.00
L	7.47	68.69	0.00	88.05

[Percent Yield]

Amino Acid	: A,V,L
Initial Yield(%)	: 880.53
Repetitive Yield(%)	: 100.00
Correlation Coef.	: 1.000
Number of Data	: 1

[Repetitive Yield(%)]

Data File : PTH-AA_08-04-2022_D01.lcd
Sample Name : PTH-AA
Method File : PTH-AA_08-04-2022.lcm
Background Data File :

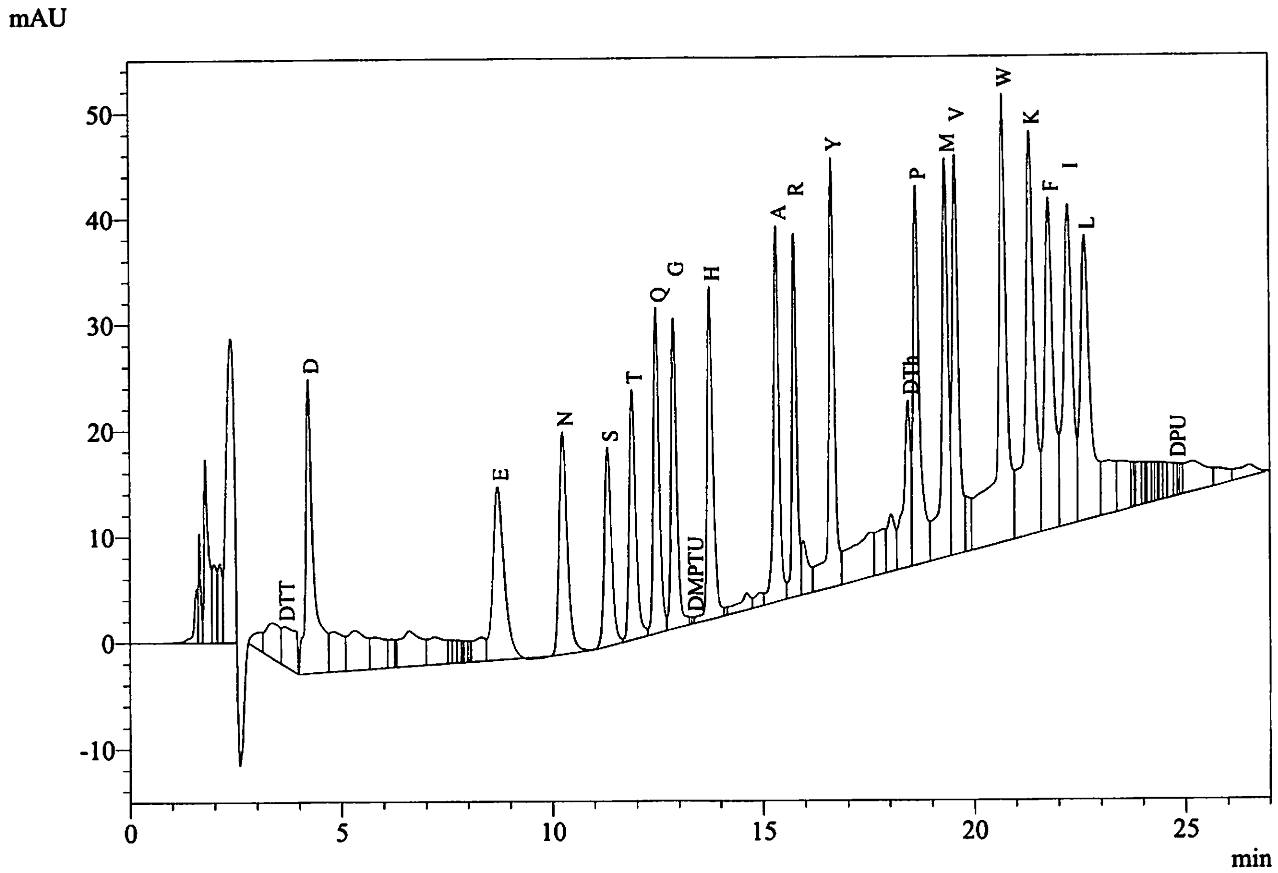

Peak Table
PDA Ch1 269nm

Peak#	Name	Ret. Time	Area	Conc.
9	DTT	3.639	86838	10.000
10	D	4.229	368538	10.000
26	E	8.709	301709	10.000
27	N	10.248	283149	10.000
28	S	11.321	219871	10.000
29	T	11.902	253806	10.000
30	Q	12.472	292815	10.000
31	G	12.889	273666	10.000
33	DMPTU	13.333	2040	10.000
34	H	13.739	303815	10.000
38	A	15.333	320725	10.000
39	R	15.757	270026	10.000
41	Y	16.643	398165	10.000
45	DTh	18.456	184729	10.000
46	P	18.650	382080	10.000
47	M	19.342	409815	10.000
48	V	19.578	389719	10.000
50	W	20.706	688571	10.000
51	K	21.353	578793	10.000
52	F	21.795	440406	10.000
53	I	22.255	445619	10.000
54	L	22.643	434982	10.000
69	DPU	24.779	16703	10.000
Total			7346580	

Data File : 10022_08-07-2022_D01.lcd
Sample Name : Sumit Mukherjee, 2
Method File : 10022_08-07-2022.lcm
Background Data File :

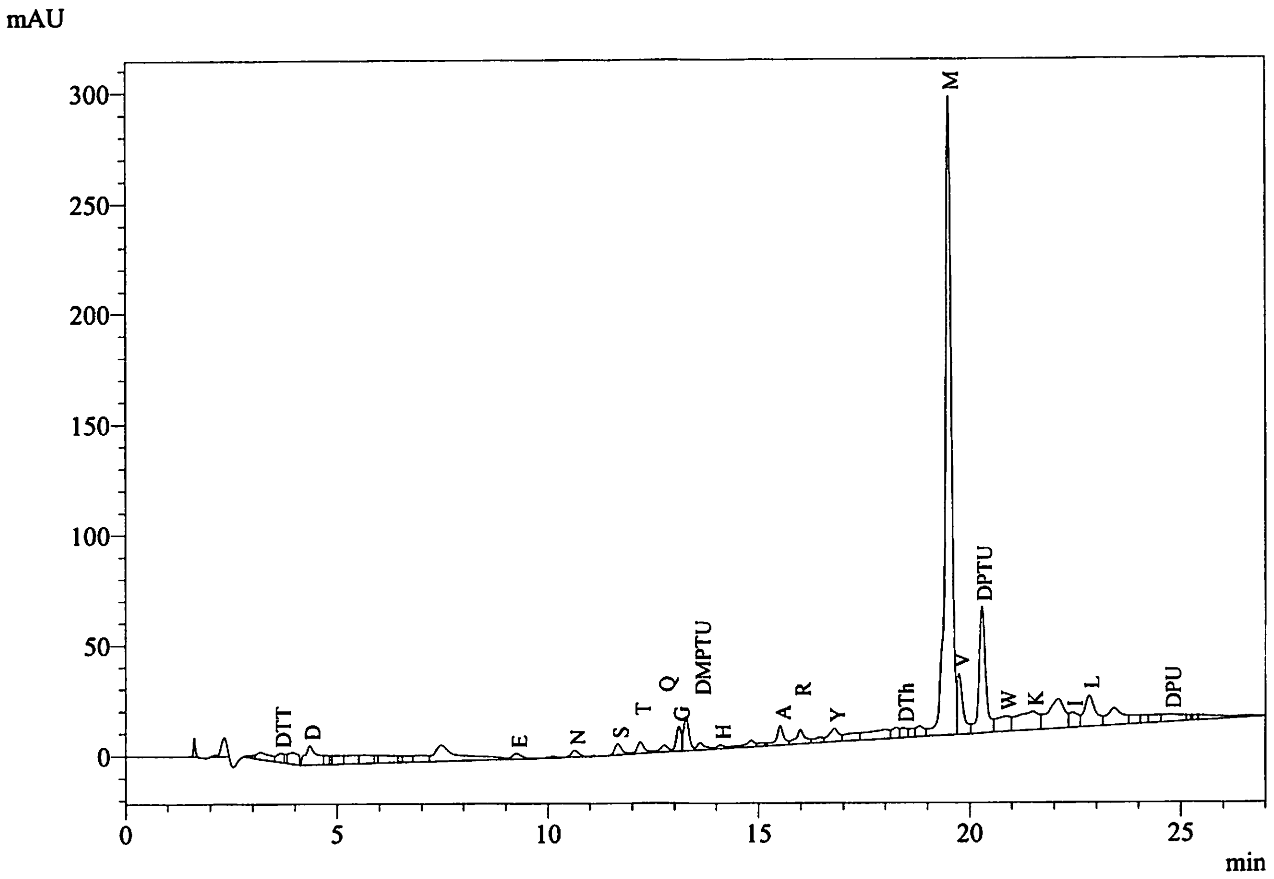

Peak Table
PDA Ch1 269nm

Peak#	Name	Ret. Time	Area	Conc.
6	DTT	3.652	49327	7.100
9	D	4.356	170151	5.771
22	E	9.248	38323	1.588
25	N	10.636	39561	1.746
27	S	11.654	59482	3.382
28	T	12.187	56008	2.758
30	Q	12.758	36545	1.560
31	G	13.099	90979	4.156
33	DMPTU	13.602	42583	260.863
34	H	14.087	21405	0.881
38	A	15.507	102028	3.976
39	R	15.983	94059	4.354
41	Y	16.792	100060	3.141
45	DTh	18.429	51909	3.513
48	M	19.511	2972116	90.654
49	V	19.747	261995	8.403
50	DPTU	20.293	638606	
51	W	20.865	160327	2.910
52	K	21.498	303176	6.548
54	I	22.434	103241	2.896
55	L	22.829	259834	7.467
60	DPU	24.758	106720	79.865
Total			5758436	

Data File	: 10022_08-07-2022_D02.lcd
Sample Name	: Sumit Mukherjee, 2
Method File	: 10022_08-07-2022.lcm
Background Data File	: 10022_08-07-2022_D01.lcd

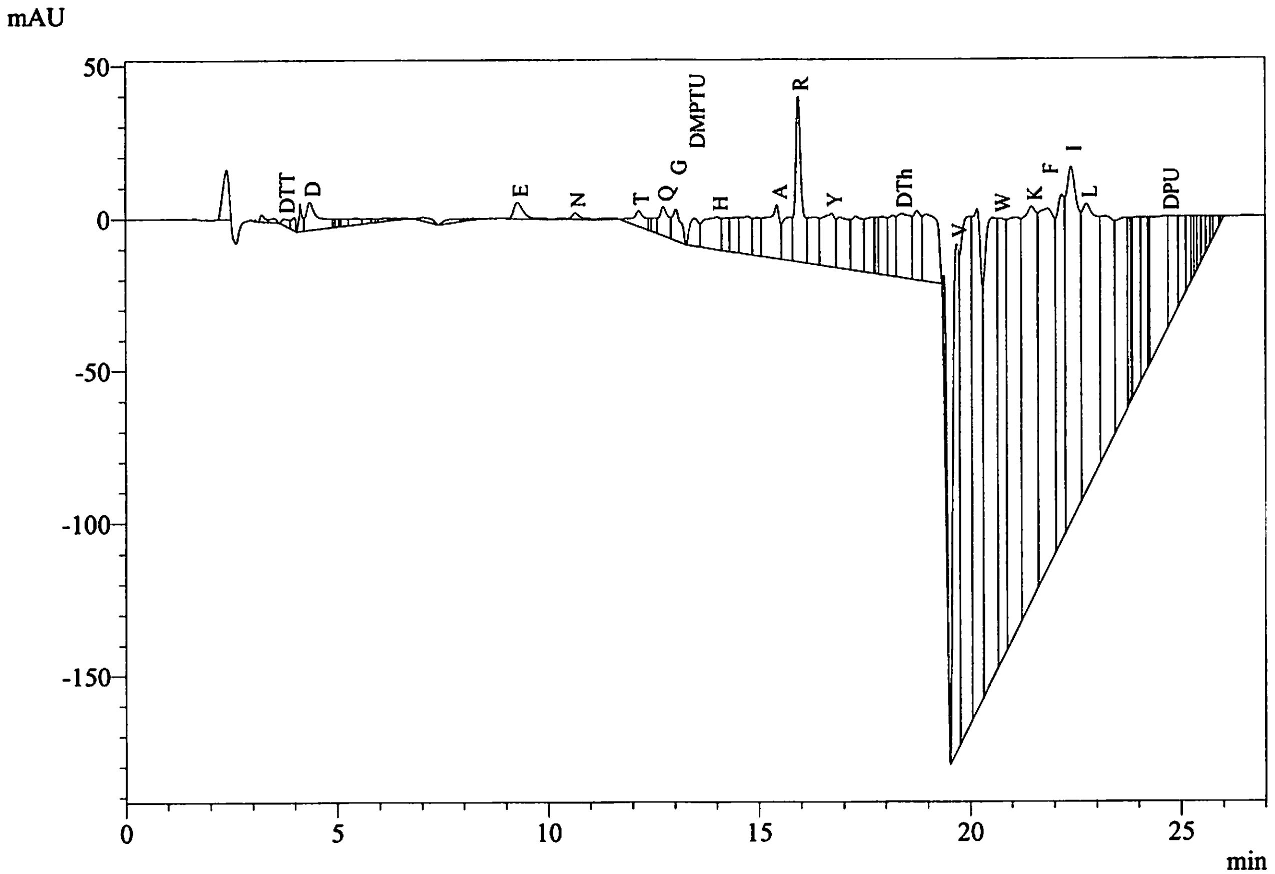

Peak Table
PDA Ch1 269nm

Peak#	Name	Ret. Time	Area	Conc.
5	DTT	3.749	28342	4.080
8	D	4.364	195321	6.625
25	E	9.285	87034	3.606
28	N	10.653	30719	1.356
33	T	12.160	103818	5.113
36	Q	12.744	145066	6.193
37	G	13.043	146327	6.684
38	DMPTU	13.485	137047	839.553
39	H	14.029	305683	12.577
44	A	15.442	395280	15.406
46	R	15.947	567572	26.274
48	Y	16.742	385043	12.088
56	DTh	18.408	469205	31.750
60	V	19.698	1694804	54.360
64	W	20.740	1799870	32.674
66	K	21.483	3038671	65.625
67	F	21.865	2928265	83.113
69	I	22.418	2444135	68.560
70	L	22.781	2390372	68.692
80	DPU	24.722	489488	366.313
Total			17782060	

Data File	: 10022_08-07-2022_D03.lcd
Sample Name	: Sumit Mukherjee, 2
Method File	: 10022_08-07-2022.lcm
Background Data File	: 10022_08-07-2022_D02.lcd

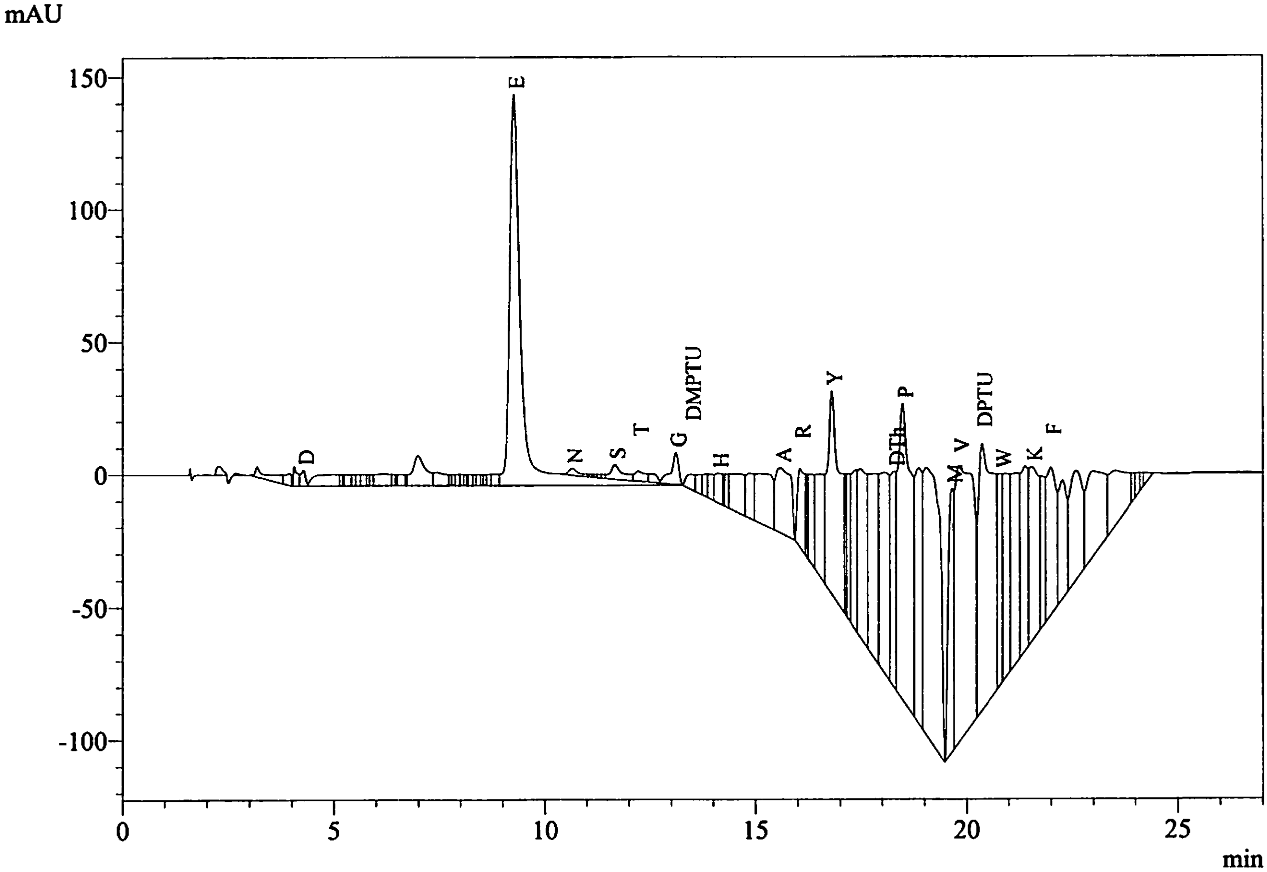

Peak Table
PDA Ch1 269nm

Peak#	Name	Ret. Time	Area	Conc.
8	D	4.284	47911	1.625
41	E	9.260	2983955	123.627
42	N	10.643	47012	2.075
49	S	11.659	116919	6.647
50	T	12.214	69625	3.429
52	G	13.099	156050	7.128
53	DMPTU	13.455	81839	501.348
57	H	14.110	140747	5.791
63	A	15.588	625413	24.375
64	R	16.051	364890	16.891
68	Y	16.801	1538837	48.310
75	DTh	18.286	718194	48.598
76	P	18.484	2404216	78.656
79	M	19.654	894345	27.279
80	V	19.822	3102215	99.502
81	DPTU	20.362	2549855	
82	W	20.818	637486	11.573
86	K	21.547	1025685	22.151
88	F	21.998	892988	25.346
Total			18398182	

Data File	: 10022_08-07-2022_D04.lcd
Sample Name	: Sumit Mukherjee, 2
Method File	: 10022_08-07-2022.lcm
Background Data File	: 10022_08-07-2022_D03.lcd

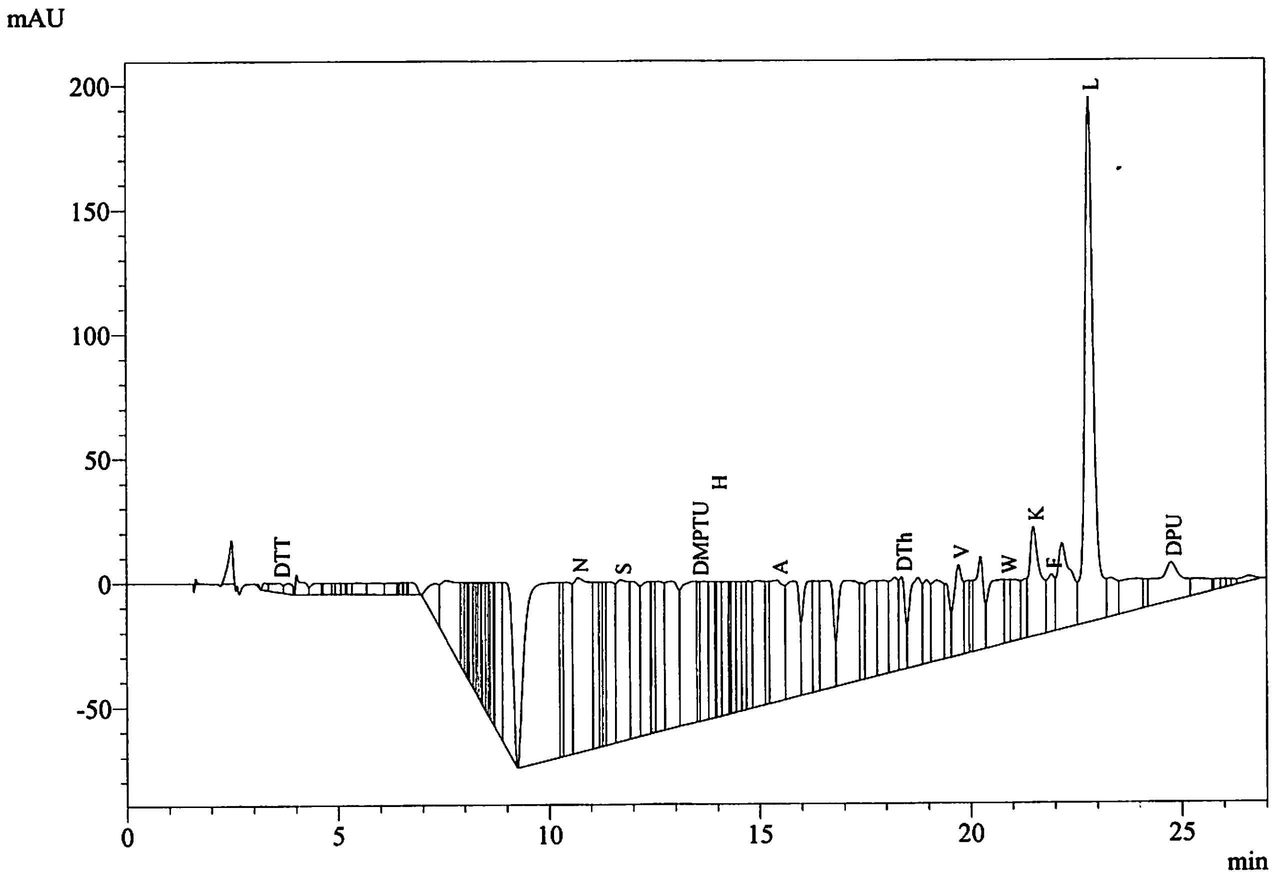

Peak Table
PDA Ch1 269nm

Peak#	Name	Ret. Time	Area	Conc.
5	DTT	3.608	72781	10.477
45	N	10.687	2039512	90.037
50	S	11.701	1315133	74.767
58	DMPTU	13.536	234324	1435.479
62	H	13.989	381983	15.716
71	A	15.424	1091083	42.524
81	DTh	18.378	367885	24.894
86	V	19.720	535926	17.189
91	W	20.862	227542	4.131
94	K	21.503	813292	17.564
95	F	21.927	288596	8.191
97	L	22.827	3064113	88.053
101	DPU	24.760	649633	486.160
Total			11081804	
