# Supplementary material for: Activation of the Plasmodium Egress Effector Subtilisin-Like Protease 1 Is Mediated by Plasmepsin X Destruction of the Prodomain
Source: mBio. 2023 Apr 10;14(2):e00673-23. doi: 10.1128/mbio.00673-23 (PMC10128010; doi:10.1128/mbio.00673-23)
Supplement: DATA SET S2 [file mbio.00673-23-s0007.pdf]

Iowa State University
Protein Facility

PROTEIN/PEPTIDE SEQUENCE REPORT

Date: August 8, 2022

To: Sumit Mukherjee

Sample Number: 10021

Sample Name: 1

Sample Preparation: The membrane was washed with DI water and loaded onto the instrument for sequence analysis.

Instrument: Shimadzu PPSQ-53A

Sequencing Method: Edman Degradation

Cycle Number	Amino Acid
1	L
2	E
3	K
4	V

The major amino acid is listed first for each cycle. If you have any questions, feel free to contact me.

Prepared by: Joel Nott

Tel: 515-294-3267, protein@iastate.edu

Protein/Peptide Sequencing Submission Form

Tracking Number-1550: 10021 Date: Aug-03-2022

Your sample ID 1

Name: SUMIT MUKHERJEE	Login: sumitmukh	Principal Investigator: Daniel Goldberg
Department/Company: Washington University School of Medicine	Phone #: 8062245077	Fax #:
E-Mail Address: sumit.mukherjee@wustl.edu	Principal Investigator E-Mail Address: dgoldberg@wustl.edu	
Mailing Address: 660 South Euclid Avenue, Department of Molecular Microbiology, Saint Louis, Missouri, 63110		
Account #: PR00136711		Assignee:
Business Purpose:		
Billing Contact Name: Rachel Warhover		
Billing Mailing Address: 4990 Children's Place, Infectious Diseases Division, Saint Louis, Missouri, 63110		
Billing Phone #: 314-454-8225	Billing E-Mail Address: rachelwarhover@wustl.edu	

✓ I agree to the terms and conditions present at http://www.biotech.iastate.edu/facilities/Agreements/ProteinTechnicalServicesAgreement.pdf

How many residues do you need? 4

Sample Information

Sample amount__________moles;or __________microgramsM.W.__________

For samples in solution: What solvent is the sample in?__________

For samples electroblotted toPVDF: What membrane was used?

____Immobilon-P (.45 micron) (Millipore) ____Problot (.1 micron) (ABI)
____Westran (.45 micron) (Schleicher & Schuell) ____Trans-Blot (.1 micron) (Biorad)
____Immobilon-PSQ (.1 micron) (Millipore) ____Fluoratrans(.1 micron) (Pall Corp)

N-Terminal blocked: No________Do not know________ Yes________

Protein/PeptideModified: Yes, at__________ with__________

Cysteine modified: Yes________ If yes, what derivative?________ No________

Enzyme treatment: Yes________ What enzyme?________ Cleavage sites________

Radioactivity: Yes________ No________

Protein sequence known: Yes______ No______ DNA sequence known: Yes______No______

Describe purification stepsin detail, especially possible contaminants such as buffer, salts, and SDS:

If your sample was collectedon an HPLC, please attachthe chromatogram with AUFS, gradient, solvents, column and wavelength.

[Sequence Analysis]

Data Acquired	:	8/7/2022 7:44:11 AM
Data Processed	:	8/8/2022 7:12:16 AM
Reactor	:	1
Number of Cycles	:	5
Sequence Schedule	:	C:\PPSQ\SeqProg3_PDA_BGE\PVDF9-3G.sch
Sample Name	:	Sumit Mukherjee, 1
Sample Amount(pmol)	:	10.0
Sample ID	:	10021
Operator Name	:	System Administrator
Data File	:	10021_08-07-2022
Start Number	:	1
Method File	:	10021_08-07-2022.lcm
Batch File	:	10021_08-07-2022.lcb
Data Folder Path	:	C:\LabSolutions\Data\Project1\PPSQ\10021_08-07-2022
Number of Analyses	:	5 / 5
Standard File	:	C:\LabSolutions\Data\Project1\PPSQ\10021_08-07-2022\PTH-AA_08-04-2022_D01.lcd
Data Comment		

[Sequence]

L E K V

[Estimated Sequence]

	1	2	3	4
1st	L	E	K	V
2nd	V	Q	G	N
3rd	M	D	N	D
4th	F	K	D	S
Reliability(%)	50.4	100.0	34.2	100.0

[Evaluated Value]

	1	2	3	4
D	0.52	111.78	74.32	51.71
E	0.04	2354.96	0.44	0.41
N	0.63	48.08	76.71	96.84
S	26.93	0.74	70.38	31.26
T	0.85	12.35	44.72	0.76
Q	0.26	120.93	0.74	1.78
G	0.83	69.72	464.85	0.60
H	3.12	0.61	0.09	1.05
A	59.62	0.78	0.93	4.59
R	0.55	53.49	0.72	0.69
Y	0.91	16.46	9.76	0.79
P	0.93	6.50	8.39	0.92
M	240.18	0.21	0.72	6.82
V	528.61	0.49	0.94	4108.36

W	0.59	2.18	3.14	0.80
K	0.81	78.69	1492.02	0.49
F	152.99	0.38	0.46	6.23
I	114.81	0.48	0.54	1.00
L	2695.89	0.31	0.23	0.94

[Amount Yield(pmol)]

	1	2	3	4
D	1.15	0.00	0.94	0.00
E	2.21	189.10	0.00	0.00
N	1.41	1.22	64.57	39.69
S	3.09	0.00	68.81	29.01
T	1.41	1.04	43.51	19.62
Q	0.79	2.30	0.00	20.67
G	5.25	1.62	73.55	26.26
H	0.00	0.00	0.00	29.43
A	4.19	0.00	43.45	28.43
R	2.79	1.55	29.18	50.19
Y	3.65	0.42	26.29	31.12
P	2.84	3.53	17.46	37.32
M	4.48	0.00	13.55	12.85
V	13.64	0.00	33.45	96.16
W	0.00	8.92	16.08	0.00
K	6.41	23.45	45.35	0.00
F	4.56	0.00	18.87	37.23
I	0.00	69.62	28.62	24.55
L	47.42	0.00	0.00	18.72

[Percent Yield]

Amino Acid	: A,V,L
Initial Yield(%)	: 374.66
Repetitive Yield(%)	: 126.57
Correlation Coef.	: 1.000
Number of Data	: 2

[Repetitive Yield(%)]

Data File : PTH-AA_08-04-2022_D01.lcd
Sample Name : PTH-AA
Method File : PTH-AA_08-04-2022.lcm
Background Data File :

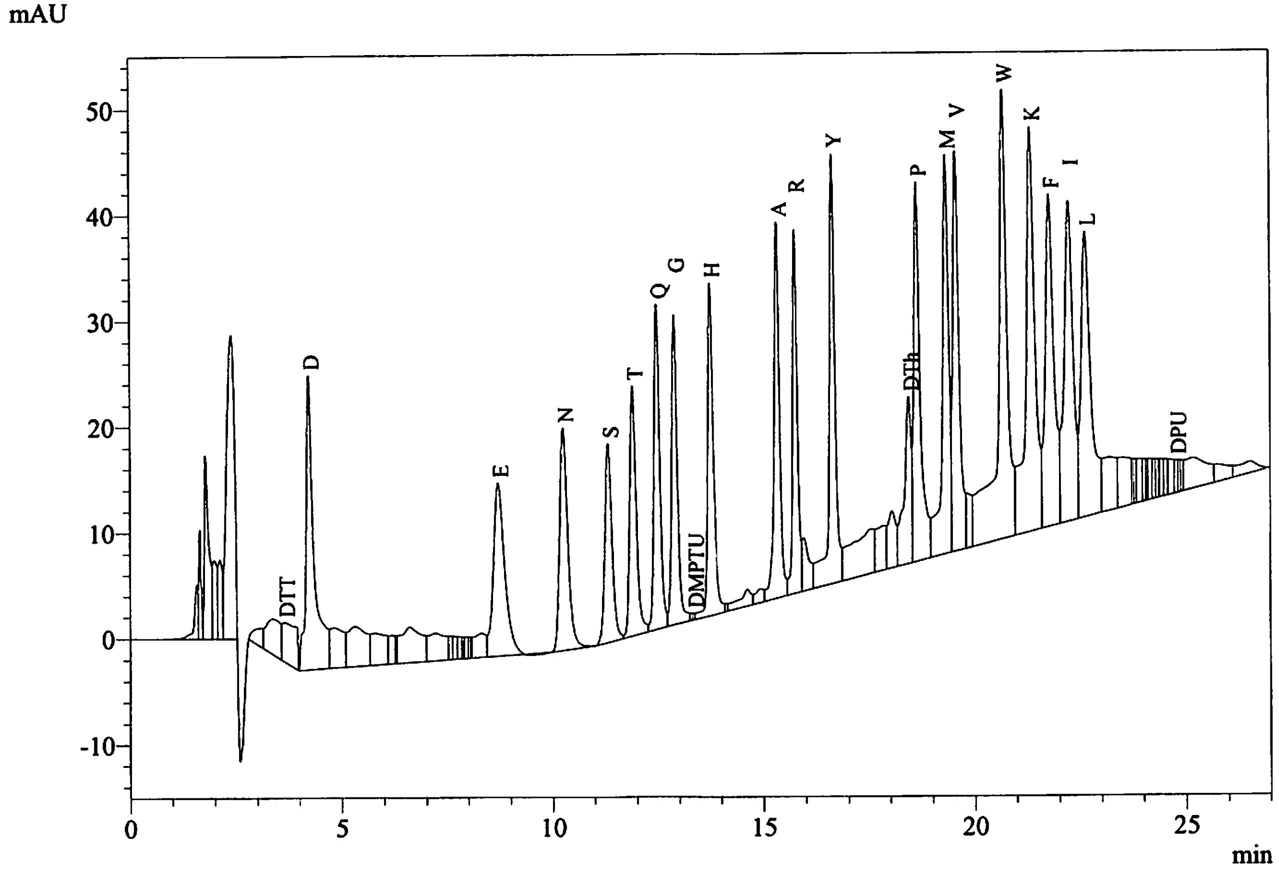

Peak Table
PDA Ch1 269nm

Peak#	Name	Ret. Time	Area	Conc.
9	DTT	3.639	86838	10.000
10	D	4.229	368538	10.000
26	E	8.709	301709	10.000
27	N	10.248	283149	10.000
28	S	11.321	219871	10.000
29	T	11.902	253806	10.000
30	Q	12.472	292815	10.000
31	G	12.889	273666	10.000
33	DMPTU	13.333	2040	10.000
34	H	13.739	303815	10.000
38	A	15.333	320725	10.000
39	R	15.757	270026	10.000
41	Y	16.643	398165	10.000
45	DTh	18.456	184729	10.000
46	P	18.650	382080	10.000
47	M	19.342	409815	10.000
48	V	19.578	389719	10.000
50	W	20.706	688571	10.000
51	K	21.353	578793	10.000
52	F	21.795	440406	10.000
53	I	22.255	445619	10.000
54	L	22.643	434982	10.000
69	DPU	24.779	16703	10.000
Total			7346580	

Data File : 10021_08-07-2022_D01.lcd
Sample Name : Sumit Mukherjee, 1
Method File : 10021_08-07-2022.lcm
Background Data File :

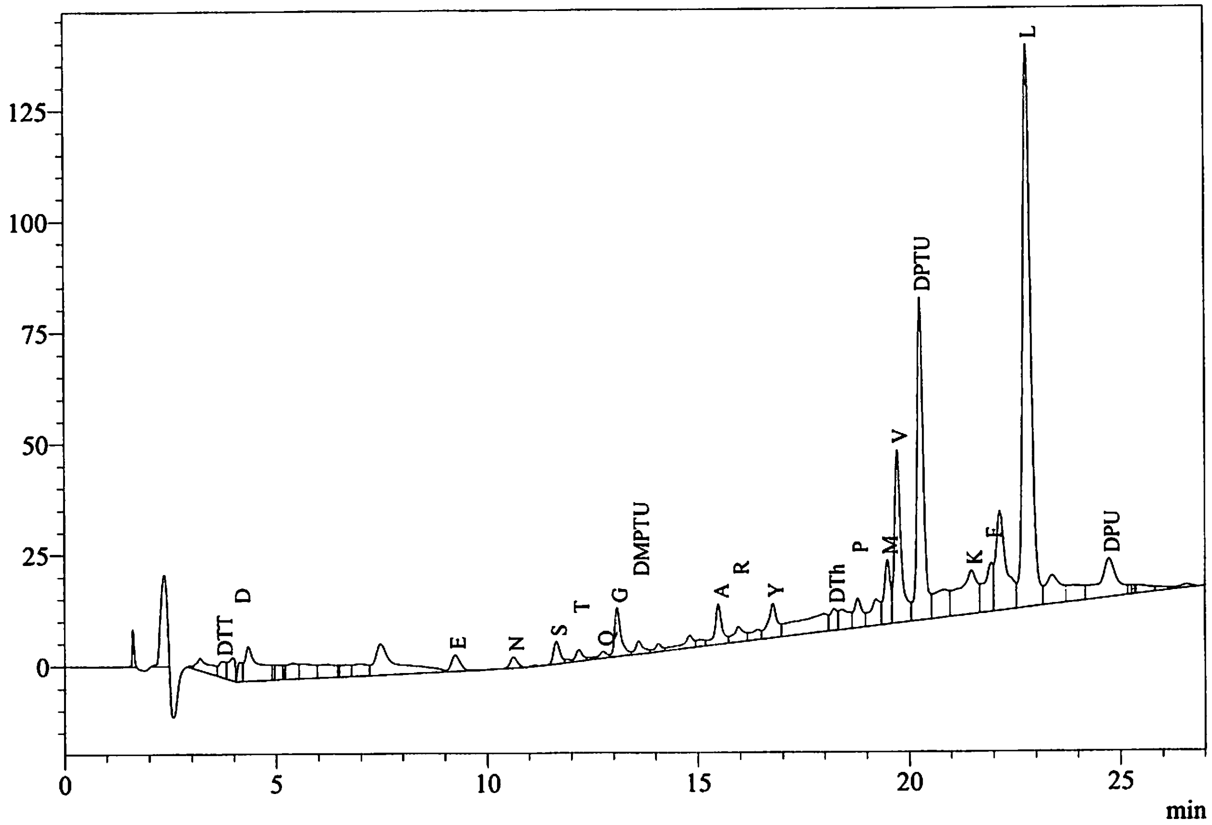

Peak Table
PDA Ch1 269nm

Peak#	Name	Ret. Time	Area	Conc.
5	DTT	3.718	42759	6.155
7	D	4.148	33902	1.150
19	E	9.237	53397	2.212
21	N	10.620	32005	1.413
23	S	11.638	54292	3.087
25	T	12.171	28601	1.409
28	Q	12.745	18609	0.794
29	G	13.077	114938	5.250
30	DMPTU	13.585	37846	231.846
34	A	15.482	107625	4.195
35	R	15.959	60355	2.794
37	Y	16.775	116167	3.647
39	DTh	18.231	57753	3.908
41	P	18.789	86806	2.840
43	M	19.490	146771	4.477
44	V	19.731	425211	13.638
45	DPTU	20.274	754594	
47	K	21.486	296851	6.411
48	F	21.955	160536	4.556
50	L	22.810	1650239	47.423
53	DPU	24.738	255113	190.916
Total			4534369	

Data File : 10021_08-07-2022_D02.lcd
Sample Name : Sumit Mukherjee, 1
Method File : 10021_08-07-2022.lcm
Background Data File : 10021_08-07-2022_D01.lcd

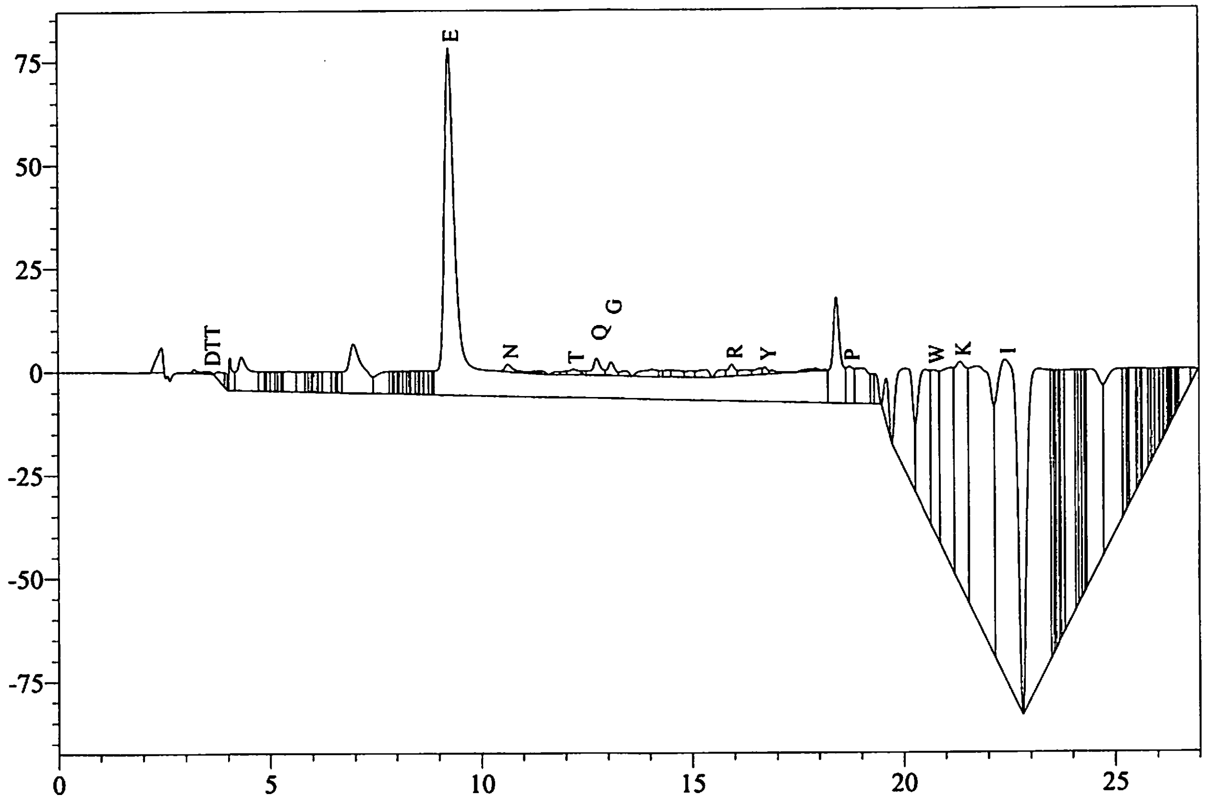

Peak Table
PDA Ch1 269nm

Peak#	Name	Ret. Time	Area	Conc.
5	DTT	3.579	2311	0.333
46	E	9.246	4564192	189.097
47	N	10.640	27685	1.222
55	T	12.188	21069	1.038
58	Q	12.746	53922	2.302
59	G	13.086	35387	1.616
68	R	15.947	33483	1.550
71	Y	16.735	13342	0.419
77	P	18.740	107918	3.531
84	W	20.744	491596	8.924
86	K	21.366	1085618	23.446
88	I	22.422	2481924	69.620
Total			8918445	

Data File	: 10021_08-07-2022_D03.lcd
Sample Name	: Sumit Mukherjee, 1
Method File	: 10021_08-07-2022.lcm
Background Data File	: 10021_08-07-2022_D02.lcd

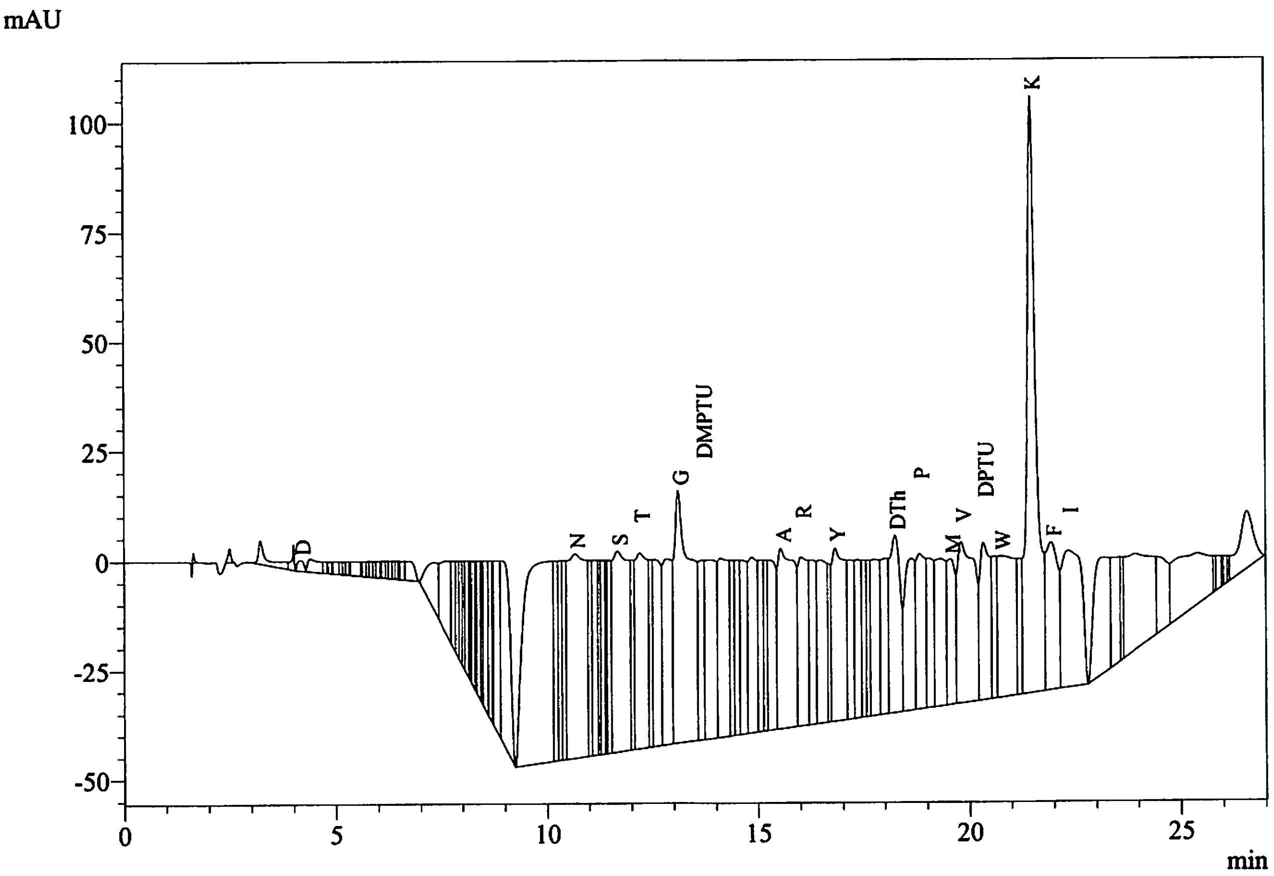

Peak Table
PDA Ch1 269nm

Peak#	Name	Ret. Time	Area	Conc.
5	D	4.160	27667	0.938
55	N	10.664	1462684	64.572
62	S	11.674	1210391	68.813
64	T	12.202	883439	43.510
68	G	13.108	1610261	73.550
69	DMPTU	13.673	394203	2414.899
79	A	15.541	1114876	43.451
80	R	16.023	630317	29.179
84	Y	16.825	837317	26.287
91	DTh	18.256	697935	47.227
93	P	18.830	533648	17.459
96	M	19.556	444101	13.546
97	V	19.812	1042949	33.452
98	DPTU	20.338	599996	
100	W	20.746	885905	16.082
102	K	21.487	2099747	45.348
103	F	21.947	664758	18.868
104	I	22.345	1020403	28.623
Total			16160598	

Data File	: 10021_08-07-2022_D04.lcd
Sample Name	: Sumit Mukherjee, 1
Method File	: 10021_08-07-2022.lcm
Background Data File	: 10021_08-07-2022_D03.lcd

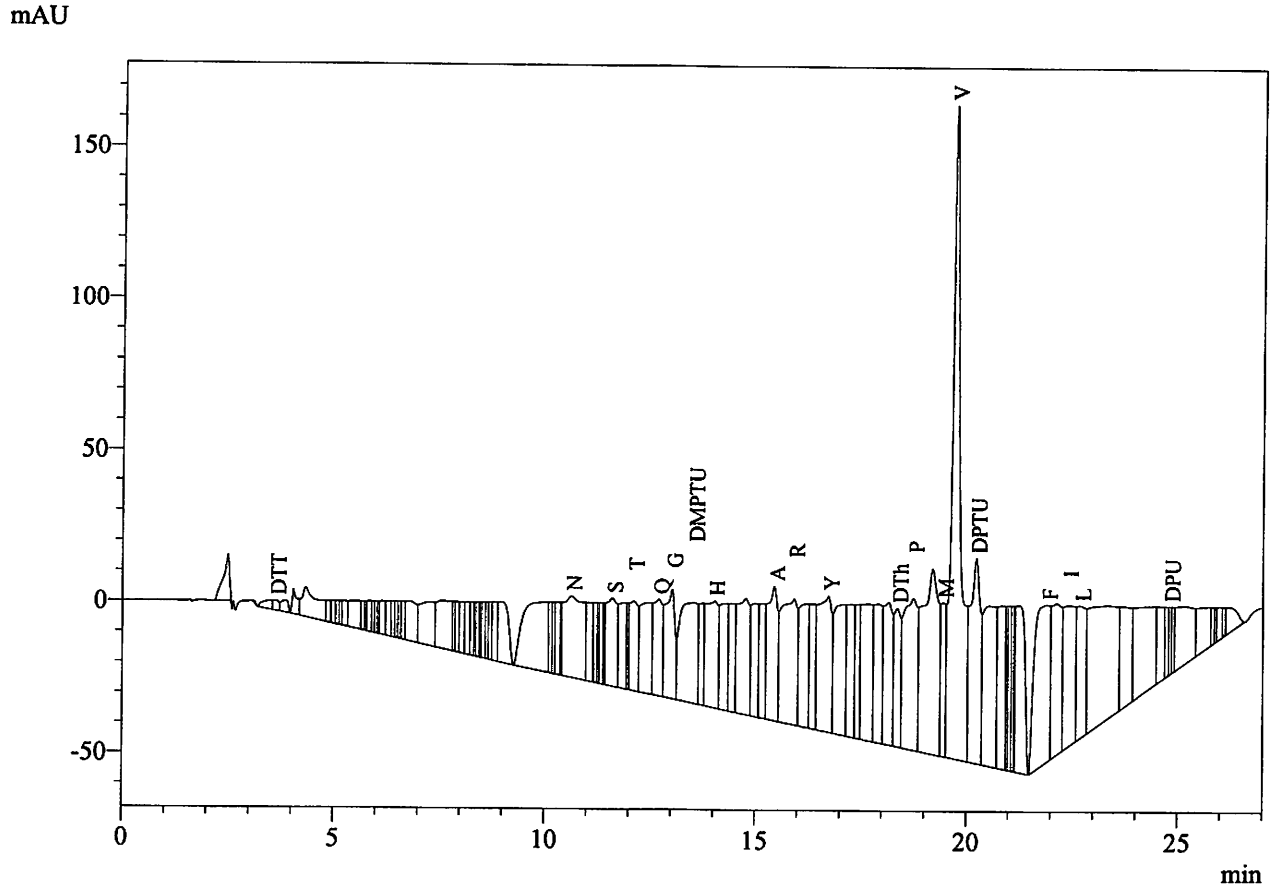

Peak Table
PDA Ch1 269nm

Peak#	Name	Ret. Time	Area	Conc.
4	DTT	3.617	31793	4.576
49	N	10.622	898963	39.686
56	S	11.601	510350	29.014
59	T	12.108	398348	19.619
61	Q	12.700	484267	20.673
62	G	13.016	574960	26.262
63	DMPTU	13.524	959796	5879.745
65	H	14.022	715344	29.432
71	A	15.442	729541	28.433
72	R	15.912	1084148	50.187
75	Y	16.724	991315	31.121
82	DTh	18.364	513028	34.715
83	P	18.742	1140819	37.323
85	M	19.445	421416	12.854
86	V	19.717	2998176	96.165
87	DPTU	20.225	1128736	
95	F	21.907	1311731	37.231
97	I	22.406	875274	24.552
98	L	22.699	651539	18.723
104	DPU	24.811	100028	74.857
Total			16519570	
