# Supplementary material for: Activation of the Plasmodium Egress Effector Subtilisin-Like Protease 1 Is Mediated by Plasmepsin X Destruction of the Prodomain
Source: mBio. 2023 Apr 10;14(2):e00673-23. doi: 10.1128/mbio.00673-23 (PMC10128010; doi:10.1128/mbio.00673-23)
Supplement: DATA SET S3 [file mbio.00673-23-s0008.pdf]

Iowa State University
Protein Facility

PROTEIN/PEPTIDE SEQUENCE REPORT

Date: August 8, 2022

To: Sumit Mukherjee

Sample Number: 10023

Sample Name: 3

Sample Preparation: The membrane was washed with DI water and loaded onto the instrument for sequence analysis.

Instrument: Shimadzu PPSQ-53A

Sequencing Method: Edman Degradation

Cycle Number	Amino Acid
1	L
2	E
3	S
4	K

The major amino acid is listed first for each cycle. If you have any questions, feel free to contact me.

Prepared by: Joel Nott
Tel: 515-294-3267, protein@iastate.edu

Protein/Peptide Sequencing Submission Form

Tracking Number-1550: 10023 Date: Aug-03-2022

Your sample ID 3

Name: SUMIT MUKHERJEE	Login: sumitmukh	Principal Investigator: Daniel Goldberg
Department/Company: Washington University School of Medicine	Phone #: 8062245077	Fax #:
E-Mail Address: sumit.mukherjee@wustl.edu	Principal Investigator E-Mail Address: dgoldberg@wustl.edu	
Mailing Address: 660 South Euclid Avenue, Department of Molecular Microbiology, Saint Louis, Missouri, 63110		
Account #: PR00136711		Assignee:
Business Purpose:		
Billing Contact Name: Rachel Warhover		
Billing Mailing Address: 4990 Children's Place, Infectious Diseases Division, Saint Louis, Missouri, 63110		
Billing Phone #: 314-454-8225	Billing E-Mail Address: rachelwarhover@wustl.edu	

✓ I agree to the terms and conditions present at http://www.biotech.iastate.edu/facilities/Agreements/ProteinTechnicalServicesAgreement.pdf

How many residues do you need? 4

Sample Information
Sample amount______moles;or ______microgramsM.W.______

For samples in solution: What solvent is the sample in?______

For samples electroblotted toPVDF: What membrane was used?

____Immobilon-P (.45 micron) (Millipore) ____Problot (.1 micron) (ABI)
____Westran (.45 micron) (Schleicher & Schuell) ____Trans-Blot (.1 micron) (Biorad)
____Immobilon-PSQ (.1 micron) (Millipore) ____Fluoratrans(.1 micron) (Pall Corp)

N-Terminal blocked: No______Do not know______ Yes______

Protein/PeptideModified: Yes, at______ with______

Cysteine modified: Yes______ If yes, what derivative?______ No______

Enzyme treatment: Yes______ What enzyme?______ Cleavage sites______

Radioactivity: Yes______ No______

Protein sequence known: Yes______ No______ DNA sequence known: Yes______No______

Describe purification stepsin detail, especially possible contaminants such as buffer, salts, and SDS:

______

______

If your sample was collectedon an HPLC, please attachthe chromatogram with AUFS, gradient, solvents, column and wavelength.

[Sequence Analysis]

Data Acquired	: 8/7/2022 5:19:52 PM
Data Processed	: 8/8/2022 7:31:33 AM
Reactor	: 3
Number of Cycles	: 5
Sequence Schedule	: C:\PPSQ\SeqProg3_PDA_BGE\PVDF9-3G.sch
Sample Name	: Sumit Mukherjee, 3
Sample Amount(pmol)	: 10.0
Sample ID	: 10023
Operator Name	: System Administrator
Data File	: 10023_08-07-2022
Start Number	: 1
Method File	: 10023_08-07-2022.lcm
Batch File	: 10023_08-07-2022.lcb
Data Folder Path	: C:\LabSolutions\Data\Project1\PPSQ\10023_08-07-2022
Number of Analyses	: 5 / 5
Standard File	: C:\LabSolutions\Data\Project1\PPSQ\10023_08-07-2022\PTH-AA_08-04-2022_D01.lcd
Data Comment	

[Sequence]

L E S K

[Estimated Sequence]

	1	2	3	4
1st	L	E	S	K
2nd	S	P	V	L
3rd	G	R	N	F
4th	D	K	W	V
Reliability(%)	27.6	26.6	71.6	100.0

[Evaluated Value]

	1	2	3	4
D	837.44	0.43	0.57	0.87
E	0.13	3794.90	0.23	0.41
N	198.26	0.39	139.46	0.63
S	2084.85	0.12	1628.95	0.31
T	420.58	0.23	0.99	23.43
Q	102.93	0.52	0.44	7.17
G	982.48	0.28	0.92	23.45
H	649.09	0.28	0.37	0.84
A	497.66	0.25	0.66	48.10
R	0.83	0.77	0.31	0.74
Y	392.68	0.37	0.77	0.90
P	0.01	1523.60	0.16	0.16
M	810.57	0.11	0.59	14.64
V	332.34	0.37	207.66	132.18

W	114.62	0.15	60.90	0.79
K	17.20	0.72	0.79	3824.95
F	601.35	0.09	0.42	235.27
I	273.73	0.37	0.65	56.28
L	5939.41	0.09	0.31	340.43

[Amount Yield(pmol)]

	1	2	3	4
D	24.49	0.00	0.00	0.00
E	12.11	249.76	0.00	0.00
N	5.94	0.00	505.66	0.00
S	42.78	0.00	306.08	0.00
T	10.31	0.00	80.89	38.21
Q	3.87	0.00	85.74	31.31
G	22.36	0.00	0.00	35.43
H	13.28	98.77	80.07	25.08
A	10.45	0.00	71.59	35.85
R	8.36	94.20	0.00	21.82
Y	8.57	0.00	30.96	16.59
P	3.17	176.70	0.00	0.00
M	14.18	0.00	0.00	9.59
V	10.67	0.00	72.85	0.00
W	5.55	0.00	71.81	3.54
K	5.89	154.00	0.00	36.12
F	10.81	60.41	23.97	5.14
I	0.00	184.12	27.84	5.96
L	83.67	0.00	0.00	13.23

[Percent Yield]

Amino Acid	: A,V,L
Initial Yield(%)	: 836.69
Repetitive Yield(%)	: 100.00
Correlation Coef.	: 1.000
Number of Data	: 1

[Repetitive Yield(%)]

Data File	: PTH-AA_08-04-2022_D01.lcd
Sample Name	: PTH-AA
Method File	: PTH-AA_08-04-2022.lcm
Background Data File	:

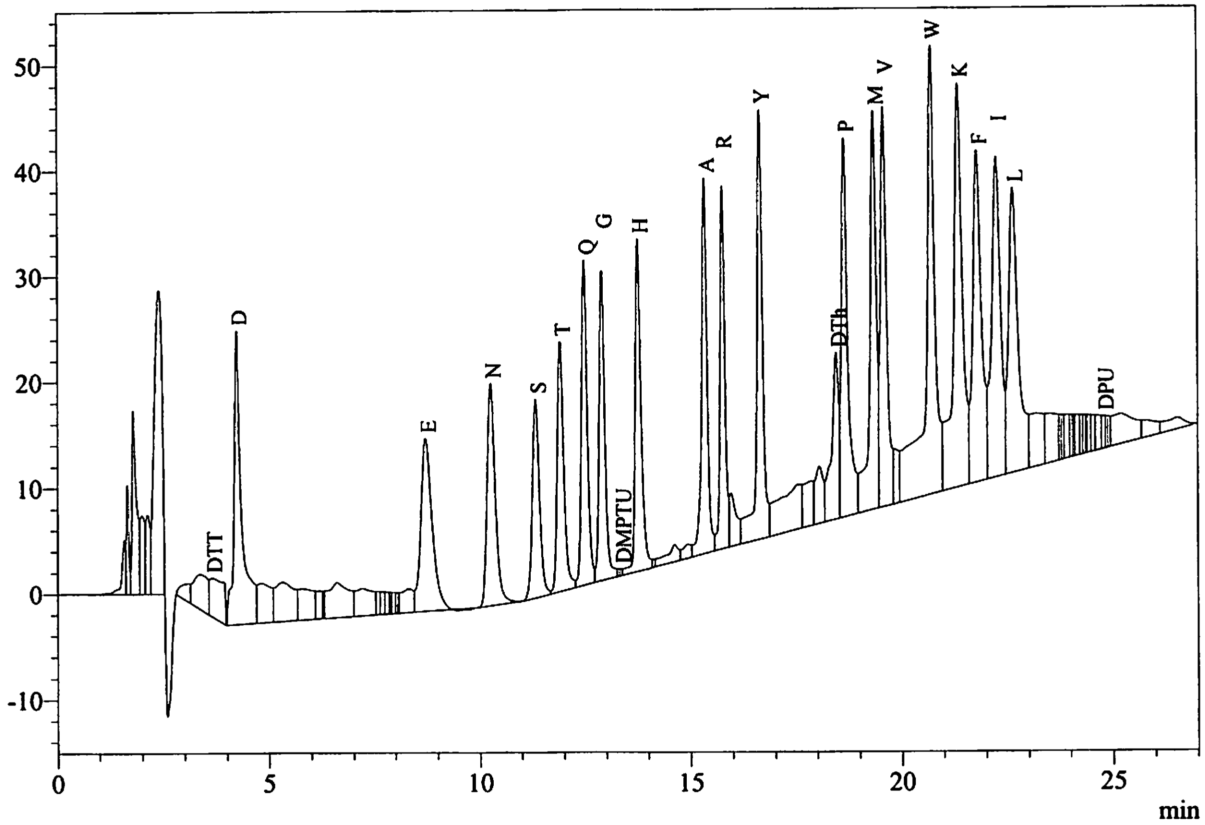

Peak Table
PDA Ch1 269nm

Peak#	Name	Ret. Time	Area	Conc.
9	DTT	3.639	86838	10.000
10	D	4.229	368538	10.000
26	E	8.709	301709	10.000
27	N	10.248	283149	10.000
28	S	11.321	219871	10.000
29	T	11.902	253806	10.000
30	Q	12.472	292815	10.000
31	G	12.889	273666	10.000
33	DMPTU	13.333	2040	10.000
34	H	13.739	303815	10.000
38	A	15.333	320725	10.000
39	R	15.757	270026	10.000
41	Y	16.643	398165	10.000
45	DTh	18.456	184729	10.000
46	P	18.650	382080	10.000
47	M	19.342	409815	10.000
48	V	19.578	389719	10.000
50	W	20.706	688571	10.000
51	K	21.353	578793	10.000
52	F	21.795	440406	10.000
53	I	22.255	445619	10.000
54	L	22.643	434982	10.000
69	DPU	24.779	16703	10.000
Total			7346580	

Data File : 10023_08-07-2022_D01.lcd
Sample Name : Sumit Mukherjee, 3
Method File : 10023_08-07-2022.lcm
Background Data File :

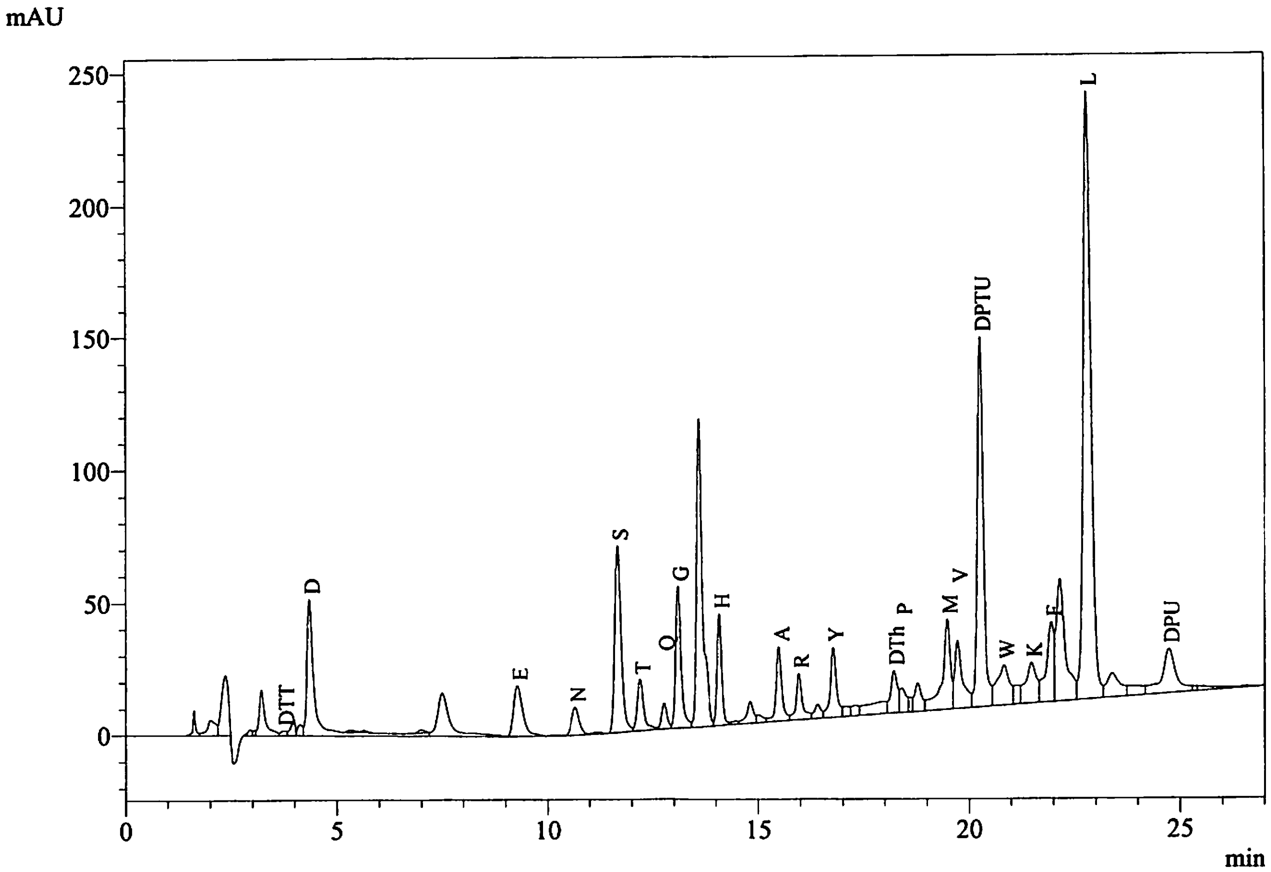

Peak Table
PDA Ch1 269nm

Peak#	Name	Ret. Time	Area	Conc.
7	DTT	3.732	18348	2.641
10	D	4.347	722107	24.492
15	E	9.273	292360	12.113
16	N	10.647	134573	5.941
18	S	11.659	752419	42.776
19	T	12.185	209336	10.310
20	Q	12.756	90735	3.873
21	G	13.086	489593	22.363
23	H	14.072	322846	13.283
27	A	15.481	268019	10.446
28	R	15.954	180593	8.360
30	Y	16.772	272914	8.568
34	DTh	18.218	179248	12.129
35	P	18.405	97031	3.174
38	M	19.479	464923	14.181
39	V	19.723	332611	10.668
40	DPTU	20.264	1388645	
41	W	20.833	305818	5.552
43	K	21.480	272746	5.890
44	F	21.946	380823	10.809
46	L	22.804	2911544	83.669
49	DPU	24.736	398288	298.063
Total			10485522	

Data File	: 10023_08-07-2022_D02.lcd
Sample Name	: Sumit Mukherjee, 3
Method File	: 10023_08-07-2022.lcm
Background Data File	: 10023_08-07-2022_D01.lcd

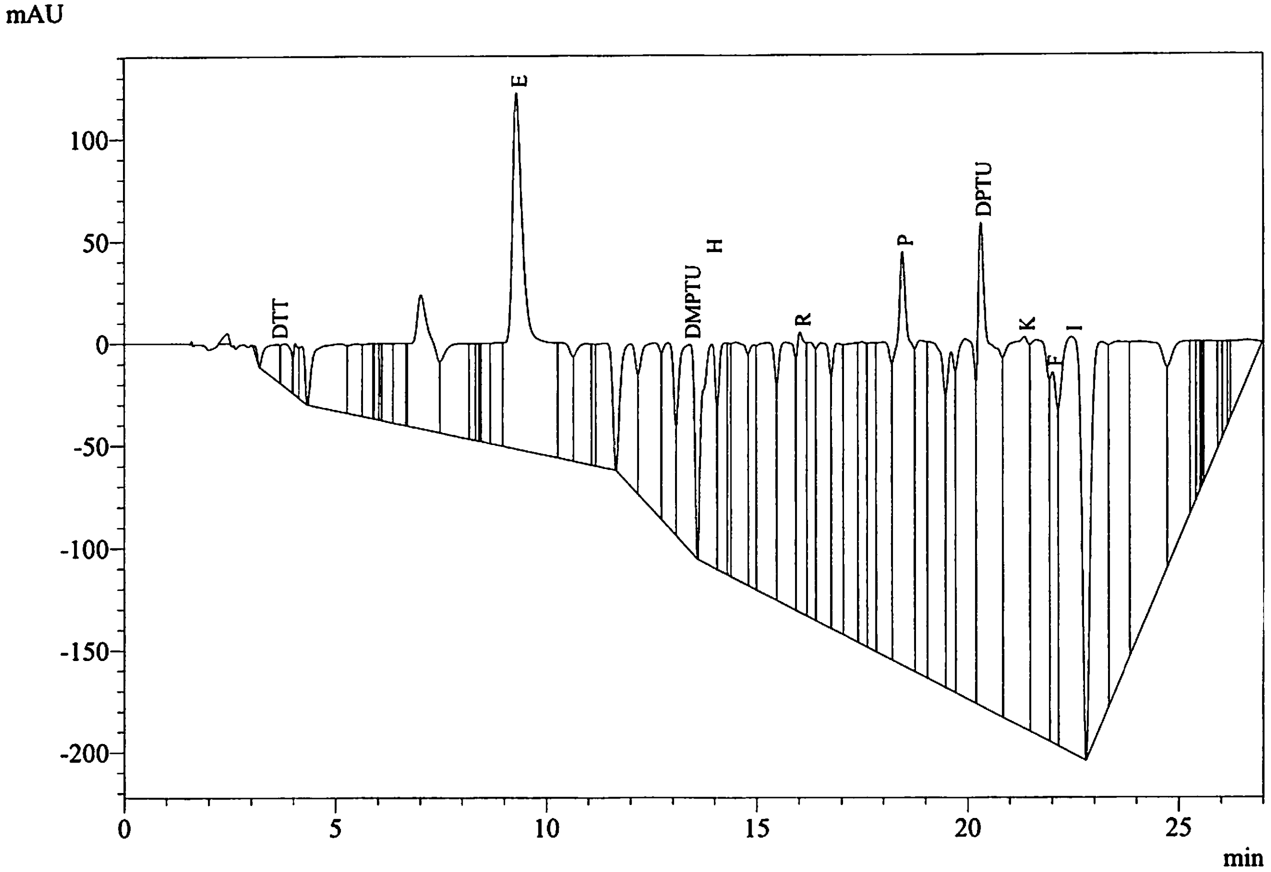

Peak Table
PDA Ch1 269nm

Peak#	Name	Ret. Time	Area	Conc.
4	DTT	3.615	385916	55.551
24	E	9.297	6028348	249.758
32	DMPTU	13.405	2427254	14869.448
33	H	13.928	2400610	98.769
40	R	16.025	2034845	94.197
48	P	18.461	5401151	176.702
53	DPTU	20.316	7228865	
54	K	21.353	7130963	154.005
56	F	22.020	2128261	60.406
57	I	22.463	6563814	184.121
Total			41730027	

Data File	: 10023_08-07-2022_D03.lcd
Sample Name	: Sumit Mukherjee, 3
Method File	: 10023_08-07-2022.lcm
Background Data File	: 10023_08-07-2022_D02.lcd

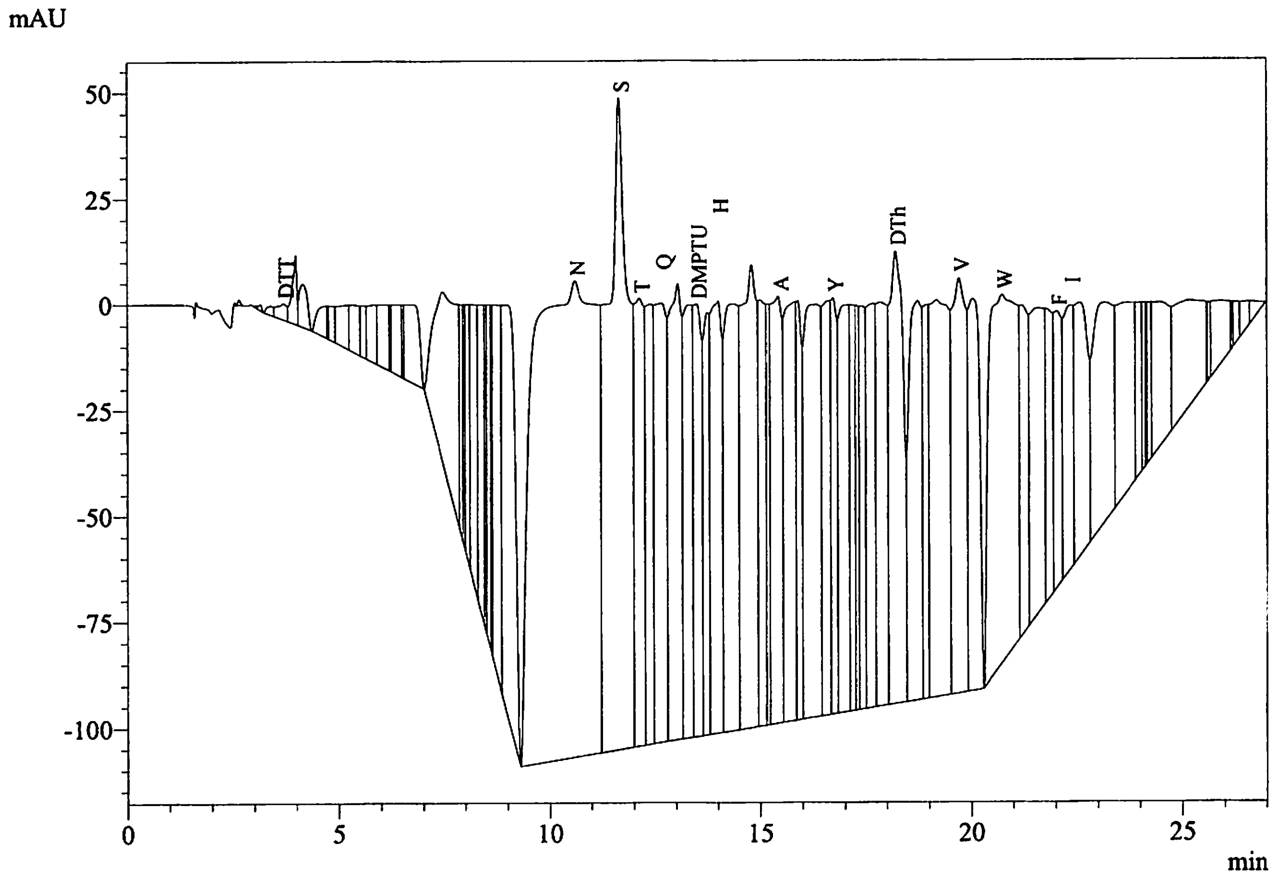

Peak Table
PDA Ch1 269nm

Peak#	Name	Ret. Time	Area	Conc.
7	DTT	3.692	62288	8.966
33	N	10.603	11454258	505.664
34	S	11.654	5383928	306.084
35	T	12.138	1642415	80.889
37	Q	12.659	2008472	85.740
40	DMPTU	13.491	1371945	8404.586
42	H	14.011	1946047	80.067
47	A	15.434	1836814	71.588
52	Y	16.732	986138	30.959
59	DTh	18.221	2425043	164.095
63	V	19.722	2271217	72.848
65	W	20.753	3955496	71.806
69	F	22.041	844417	23.967
70	I	22.356	992345	27.836
Total			37180820	

Data File	: 10023_08-07-2022_D04.lcd
Sample Name	: Sumit Mukherjee, 3
Method File	: 10023_08-07-2022.lcm
Background Data File	: 10023_08-07-2022_D03.lcd

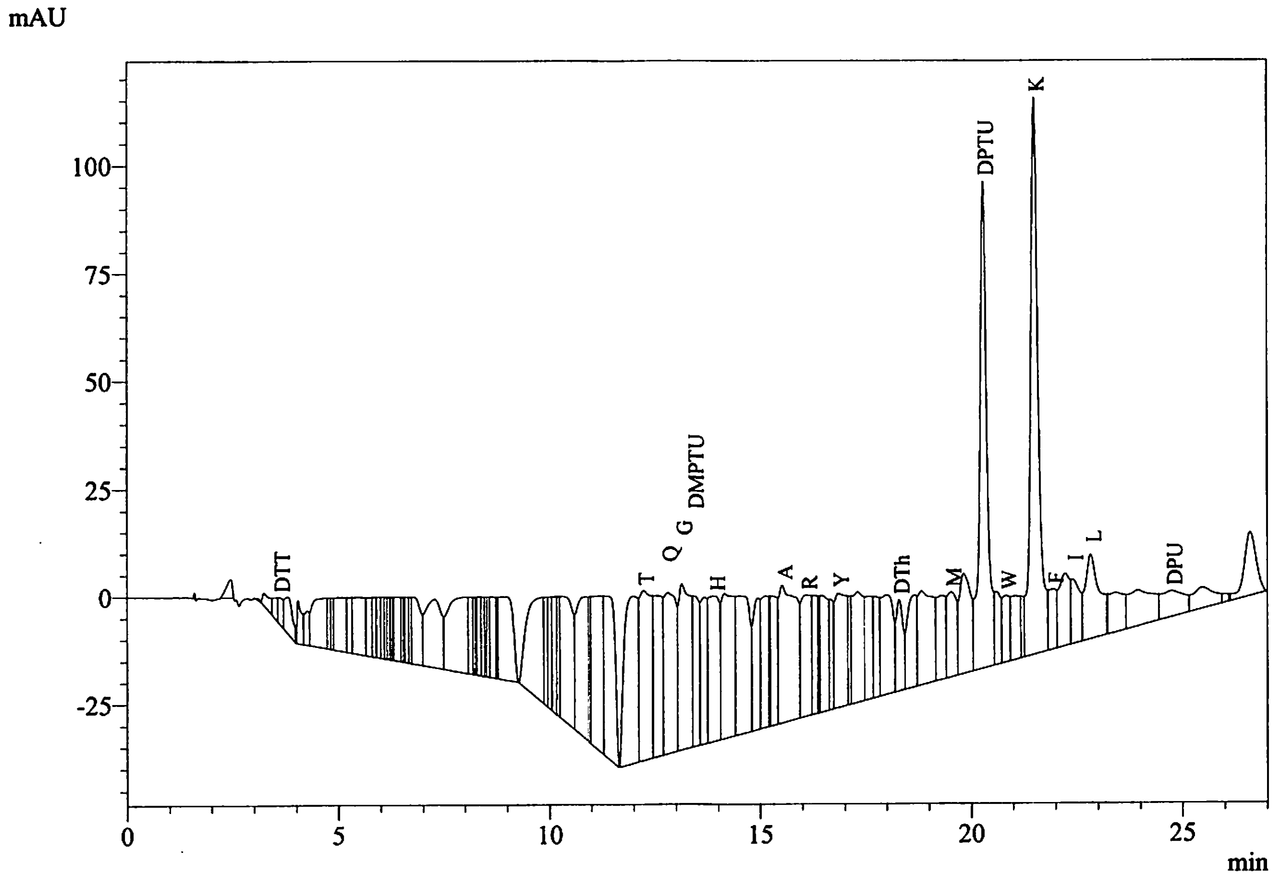

Peak Table
PDA Ch1 269nm

Peak#	Name	Ret. Time	Area	Conc.
6	DTT	3.599	50161	7.221
53	T	12.232	775846	38.211
55	Q	12.805	733546	31.314
56	G	13.137	775681	35.430
57	DMPTU	13.416	363001	2223.760
59	H	13.920	609648	25.083
66	A	15.523	919927	35.853
67	R	16.102	471411	21.822
72	Y	16.853	528602	16.595
78	DTh	18.301	245635	16.621
82	M	19.525	314338	9.588
84	DPTU	20.292	1373452	
86	W	20.835	194822	3.537
89	K	21.505	1672692	36.125
90	F	21.945	181224	5.144
92	I	22.413	212437	5.959
93	L	22.832	460558	13.235
97	DPU	24.755	228104	170.704
Total			10111086	
