# Supplementary material for: Activation of the Plasmodium Egress Effector Subtilisin-Like Protease 1 Is Mediated by Plasmepsin X Destruction of the Prodomain
Source: mBio. 2023 Apr 10;14(2):e00673-23. doi: 10.1128/mbio.00673-23 (PMC10128010; doi:10.1128/mbio.00673-23)
Supplement: DATA SET S4 [file mbio.00673-23-s0009.pdf]

Iowa State University
Protein Facility

PROTEIN/PEPTIDE SEQUENCE REPORT

Date: September 3, 2022

To: Sumit Mukherjee

Sample Number: 10048

Sample Name: Sample 1

Sample Preparation: The membrane was washed with DI water and loaded onto the instrument for sequence analysis.

Instrument: Shimadzu PPSQ-53A

Sequencing Method: Edman Degradation

Cycle Number	Amino Acid
1	F
2	Q
3	E
4	S

The major amino acid is listed first for each cycle. If you have any questions, feel free to contact me.

Prepared by: Joel Nott
Tel: 515-294-3267, protein@iastate.edu

Protein/Peptide Sequencing Submission Form

Tracking Number-1583: 10048 Date: Sep-01-2022

Your sample ID Sample 1

Name: SUMIT MUKHERJEE	Login: sumitmukh	Principal Investigator: Daniel Goldberg
Department/Company: Washington University School of Medicine	Phone #: 8062245077	Fax #:
E-Mail Address: sumit.mukherjee@wustl.edu	Principal Investigator E-Mail Address: dgoldberg@wustl.edu	
Mailing Address: 660 South Euclid Avenue, Department of Molecular Microbiology, Saint Louis, Missouri, 63110		
Account #: PR00136711		Assignee:
Business Purpose:		
Billing Contact Name: Rachel Warhover		
Billing Mailing Address: 4990 Children's Place, Infectious Diseases Division, Saint Louis, Missouri, 63110		
Billing Phone #: 314-454-8225	Billing E-Mail Address: rachelwarhover@wustl.edu	

✓ I agree to the terms and conditions present at http://www.biotech.iastate.edu/facilities/Agreements/ProteinTechnicalServicesAgreement.pdf

How many residues do you need? 4

Sample Information
Sample amount________moles;or ________microgramsM.W.________

For samples in solution: What solvent is the sample in?________

For samples electroblotted toPVDF: What membrane was used?
____Immobilon-P (.45 micron) (Millipore) ____Problot (.1 micron) (ABI)
____Westran (.45 micron) (Schleicher & Schuell) ____Trans-Blot (.1 micron) (Biorad)
____Immobilon-PSQ (.1 micron) (Millipore) ____Fluoratrans(.1 micron) (Pall Corp)

N-Terminal blocked: No________Do not know________ Yes________

Protein/PeptideModified: Yes, at________ with________

Cysteine modified: Yes________ If yes, what derivative?________ No________

Enzyme treatment: Yes________ What enzyme?________ Cleavage sites________

Radioactivity: Yes________ No________

Protein sequence known: Yes______ No______ DNA sequence known: Yes______No______

Describe purification stepsin detail, especially possible contaminants such as buffer, salts, and SDS:

If your sample was collectedon an HPLC, please attachthe chromatogram with AUFS, gradient, solvents, column and wavelength.

[Sequence Analysis]

Data Acquired	:	9/2/2022 11:54:53 AM
Data Processed	:	9/2/2022 5:03:18 PM
Reactor	:	1
Number of Cycles	:	5
Sequence Schedule	:	C:\PPSQ\SeqProg3_PDA_BGE\PVDF9-3G.sch
Sample Name	:	Sumit Mukherjee, Sample 1
Sample Amount(pmol)	:	10.0
Sample ID	:	10048
Operator Name	:	System Administrator
Data File	:	10048_09-02-2022
Start Number	:	1
Method File	:	10048_09-02-2022.lcm
Batch File	:	10048_09-02-2022.lcb
Data Folder Path	:	C:\LabSolutions\Data\Project1\PPSQ\10048_09-02-2022
Number of Analyses	:	5 / 5
Standard File	:	C:\LabSolutions\Data\Project1\PPSQ\10048_09-02-2022\PTH-AA_08-31-2022_D01.lcd
Data Comment		

[Sequence]

F Q E S

[Estimated Sequence]

	1	2	3	4
1st	F	Q	E	S
2nd	M	Y	Y	A
3rd	L	E	P	I
4th	G	A	D	N
Reliability(%)	100.0	71.3	49.2	100.0

[Evaluated Value]

	1	2	3	4
D	0.49	67.06	112.94	80.60
E	0.09	958.89	6047.47	0.16
N	0.72	35.44	97.61	85.88
S	0.13	0.46	0.29	2700.62
T	0.61	37.82	66.43	0.77
Q	0.00	12323.40	0.12	0.18
G	8.28	0.79	0.95	0.87
H	0.54	0.48	0.64	0.00
A	0.32	230.92	0.76	123.47
R	1.47	0.72	0.00	1.72
Y	0.17	1689.43	1346.08	0.23
P	0.47	81.09	127.78	0.59
M	112.95	0.35	47.74	23.10
V	0.51	97.90	77.34	0.66

W	3.64	0.75	1.57	5.03
K	0.70	26.08	94.56	0.70
F	8298.50	0.09	0.25	0.76
I	0.73	30.85	56.92	91.68
L	19.74	0.80	75.51	0.94

[Amount Yield(pmol)]

	1	2	3	4
D	2.50	3.48	5.06	14.54
E	2.30	35.46	195.36	0.00
N	1.78	4.80	5.82	665.84
S	3.30	0.00	0.00	431.17
T	1.43	4.84	221.79	0.00
Q	0.83	142.42	0.00	0.00
G	10.01	1.07	358.31	197.96
H	0.50	0.18	86.63	46.41
A	2.40	2.94	107.89	103.95
R	0.00	0.00	0.00	27.71
Y	5.56	21.31	97.09	0.00
P	2.96	0.00	0.00	0.00
M	7.01	0.00	85.08	28.92
V	3.51	9.37	51.17	29.54
W	3.15	0.00	14.67	0.00
K	5.81	204.49	113.17	16.82
F	151.32	0.00	0.00	21.29
I	0.38	300.88	57.25	30.68
L	3.68	0.00	101.29	28.61

[Percent Yield]

Amino Acid	:	A,V,L
Initial Yield(%)	:	0.00
Repetitive Yield(%)	:	0.00
Correlation Coef.	:	0.000
Number of Data	:	0

[Repetitive Yield(%)]

Data File : PTH-AA_08-31-2022_D01.lcd
Sample Name : PTH-AA
Method File : PTH-AA_08-31-2022.lcm
Background Data File :

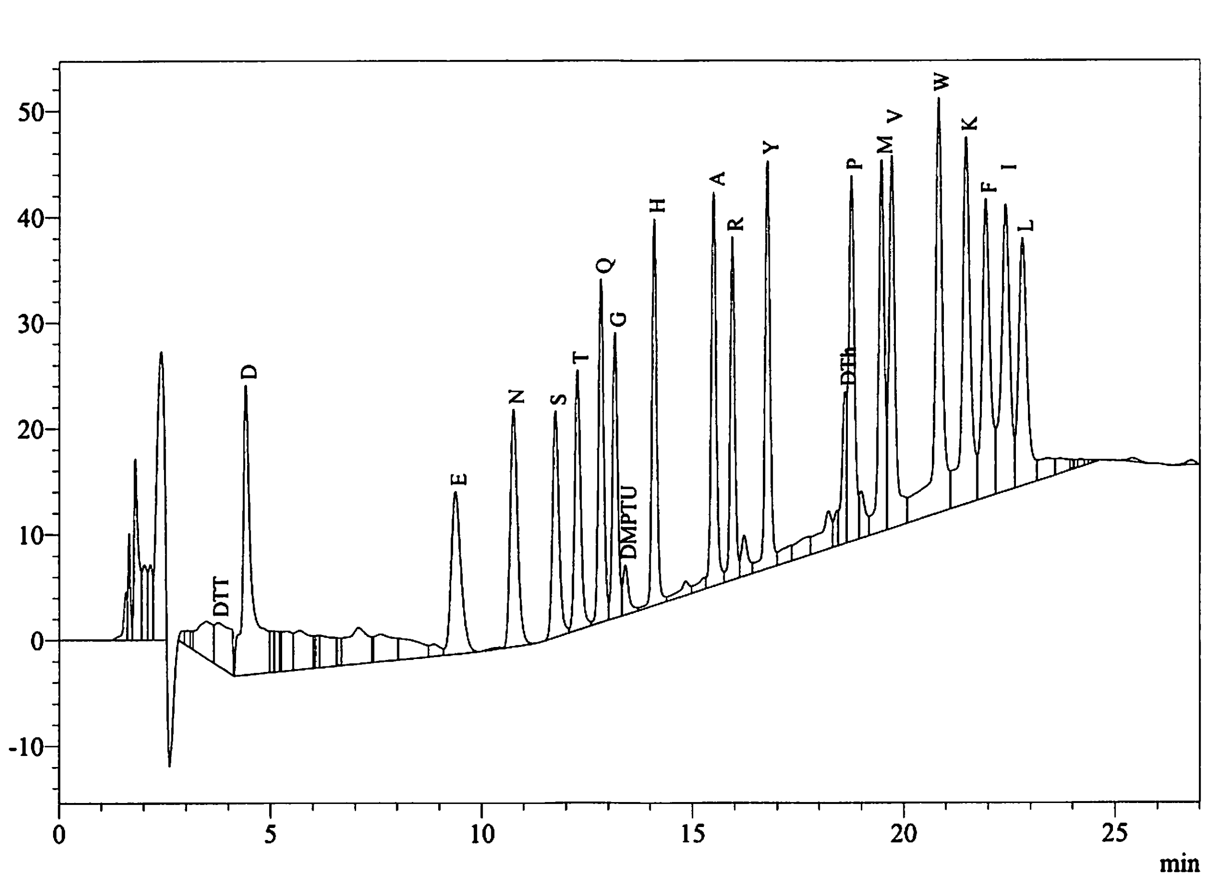

Peak Table
PDA Ch1 269nm

Peak#	Name	Ret. Time	Area	Conc.
11	DTT	3.743	113234	10.000
12	D	4.411	406405	10.000
28	E	9.370	248612	10.000
31	N	10.753	271970	10.000
32	S	11.744	226315	10.000
33	T	12.262	239710	10.000
34	Q	12.817	280338	10.000
35	G	13.148	228667	10.000
36	DMPTU	13.397	45273	10.000
37	H	14.089	285627	10.000
40	A	15.493	307920	10.000
41	R	15.935	244091	10.000
43	Y	16.766	325871	10.000
48	DTh	18.614	110915	10.000
49	P	18.763	321335	10.000
51	M	19.471	338785	10.000
52	V	19.711	362583	10.000
53	W	20.830	525709	10.000
54	K	21.476	465873	10.000
55	F	21.940	369418	10.000
56	I	22.410	380874	10.000
57	L	22.804	326467	10.000
Total			6425995	

Data File	: 10048_09-02-2022_D01.lcd
Sample Name	: Sumit Mukherjee, Sample 1
Method File	: 10048_09-02-2022.lcm
Background Data File	:

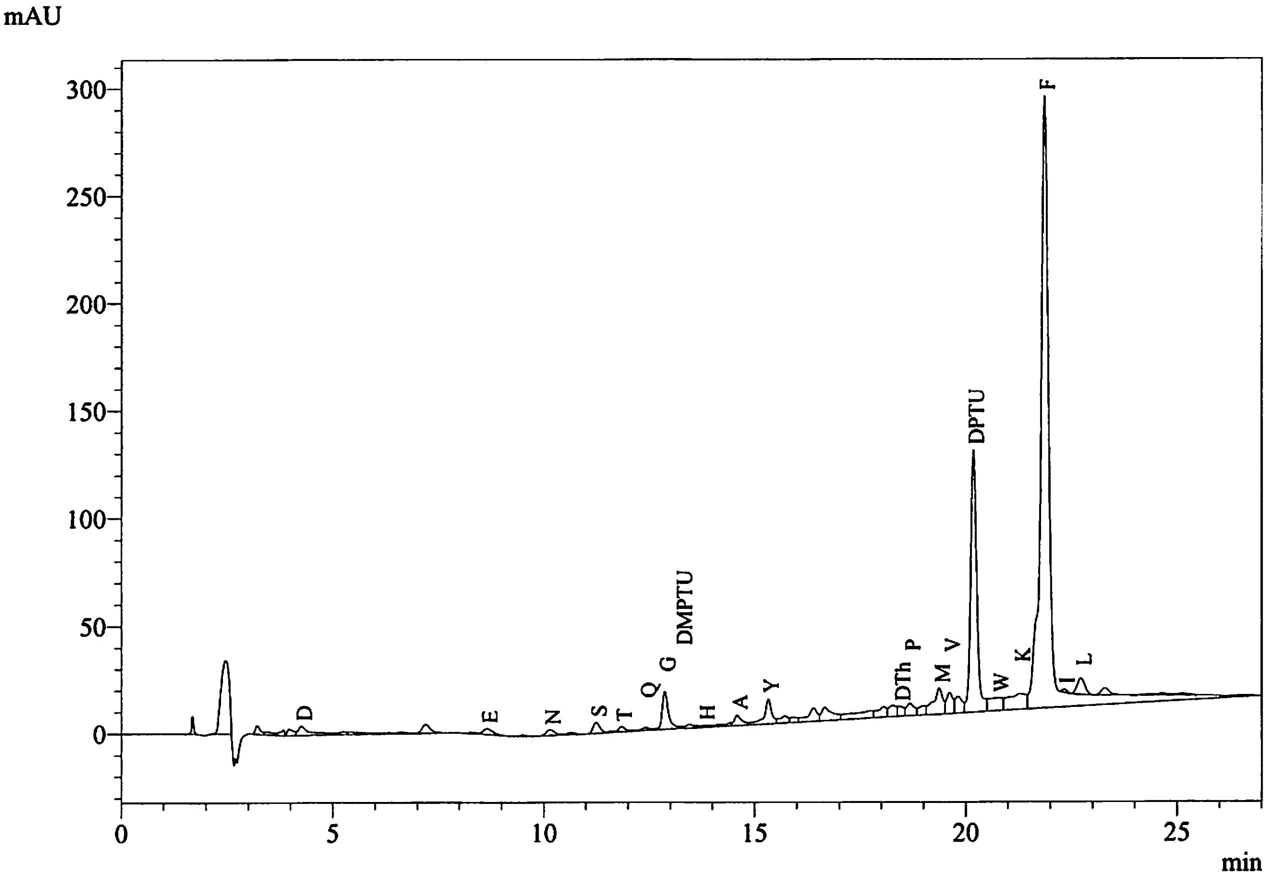

Peak Table
PDA Ch1 269nm

Peak#	Name	Ret. Time	Area	Conc.
7	D	4.256	81256	2.499
18	E	8.653	45759	2.301
20	N	10.139	38748	1.781
23	S	11.239	59708	3.298
25	T	11.838	27500	1.434
26	Q	12.412	18606	0.830
28	G	12.862	183194	10.014
29	DMPTU	13.254	7984	2.204
32	H	13.809	11406	0.499
35	A	14.583	59123	2.400
36	Y	15.323	144952	5.560
44	DTh	18.441	46191	5.206
45	P	18.678	75980	2.956
47	M	19.370	189979	7.010
48	V	19.614	101787	3.509
50	DPTU	20.177	1231602	
51	W	20.775	132658	3.154
52	K	21.278	216631	5.813
53	F	21.868	4471988	151.318
54	I	22.329	11645	0.382
55	L	22.723	96238	3.685
Total			7252933	

Data File	: 10048_09-02-2022_D02.lcd
Sample Name	: Sumit Mukherjee, Sample 1
Method File	: 10048_09-02-2022.lcm
Background Data File	: 10048_09-02-2022_D01.lcd

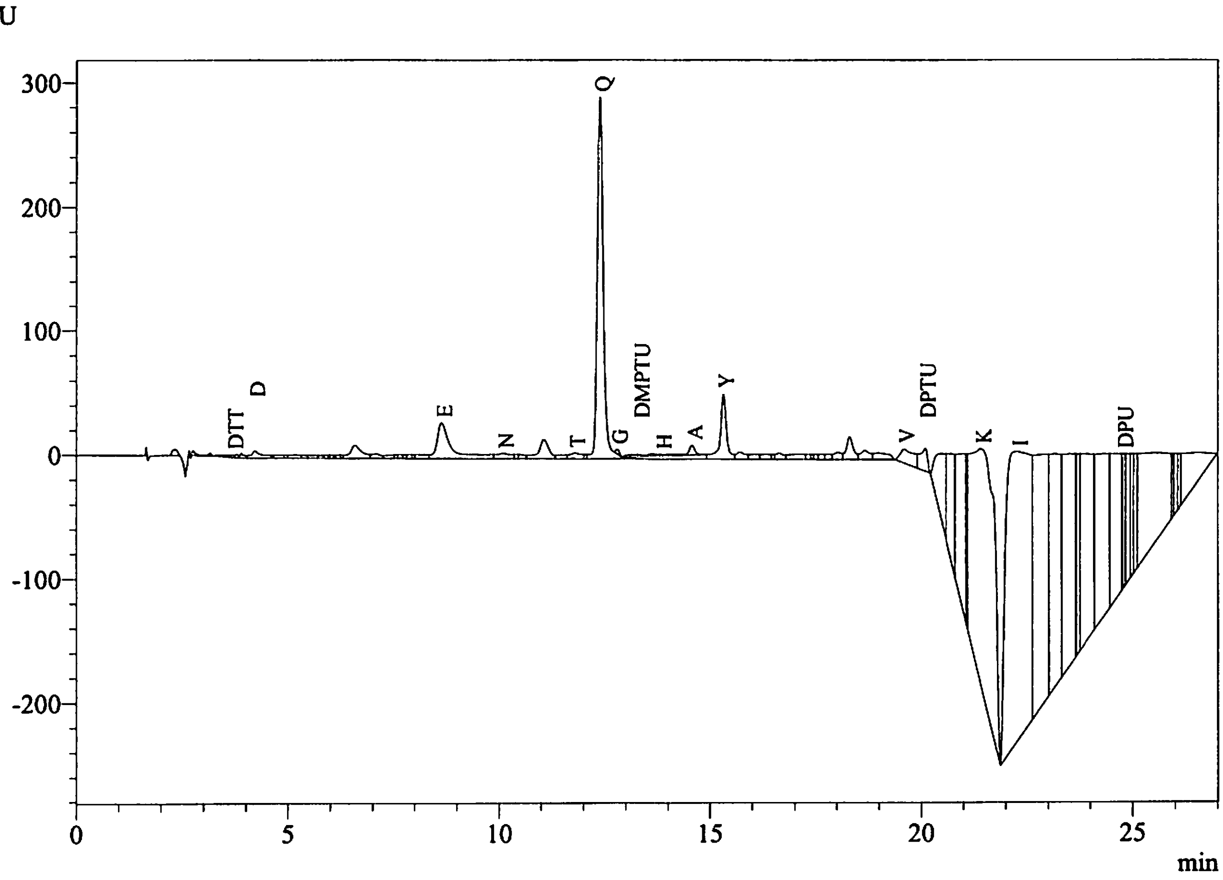

Peak Table
PDA Ch1 269nm

Peak#	Name	Ret. Time	Area	Conc.
10	DTT	3.688	15384	1.698
13	D	4.214	113023	3.476
36	E	8.636	705256	35.460
37	N	10.089	104526	4.804
42	T	11.794	92723	4.835
44	Q	12.386	3194107	142.422
45	G	12.795	19618	1.072
47	DMPTU	13.318	11789	3.255
49	H	13.840	4165	0.182
54	A	14.570	72479	2.942
55	Y	15.318	555484	21.308
71	V	19.594	271928	9.375
72	DPTU	20.095	224176	
77	K	21.410	7621402	204.492
78	I	22.271	9167650	300.875
87	DPU	24.768	314724	
Total			22488435	

Data File	: 10048_09-02-2022_D03.lcd
Sample Name	: Sumit Mukherjee, Sample 1
Method File	: 10048_09-02-2022.lcm
Background Data File	: 10048_09-02-2022_D02.lcd

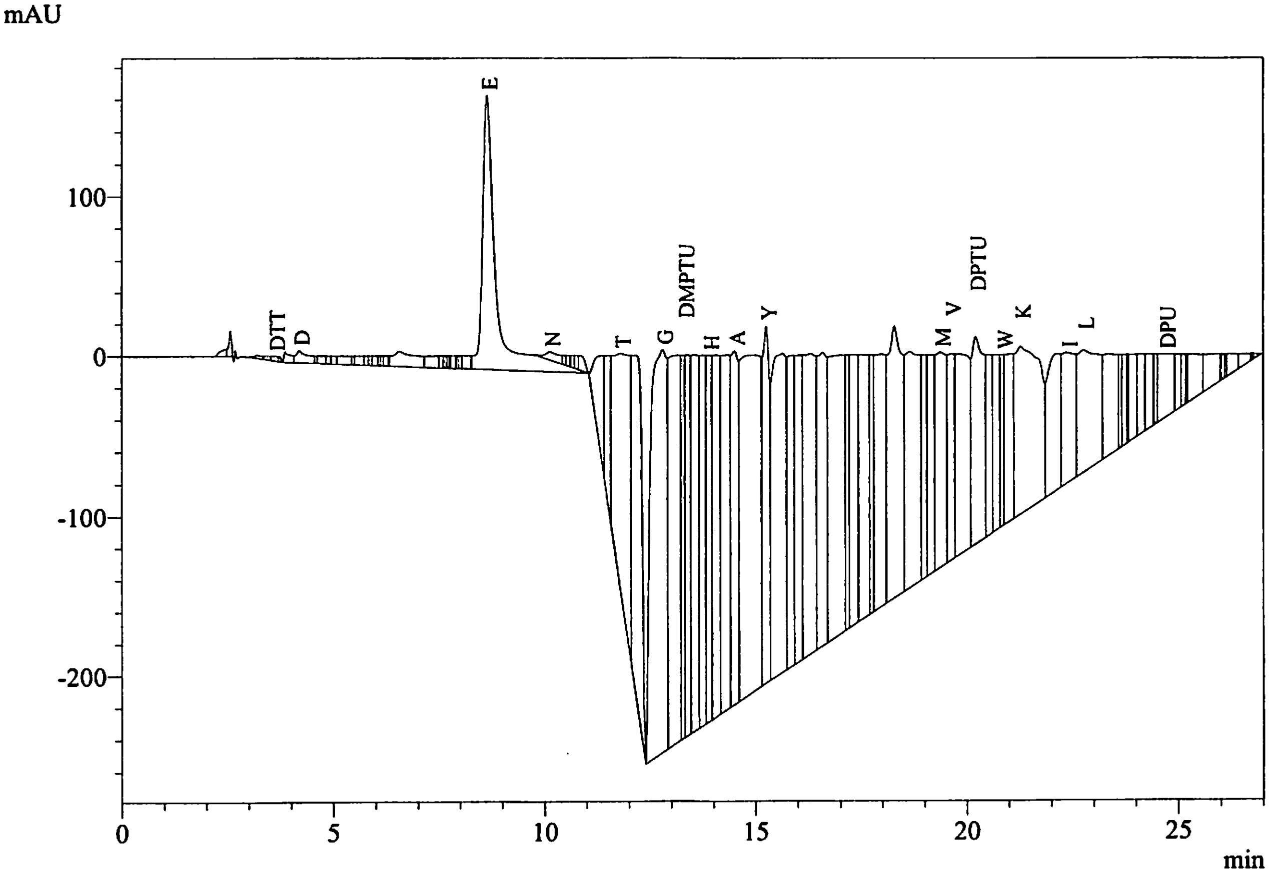

Peak Table
PDA Ch1 269nm

Peak#	Name	Ret. Time	Area	Conc.
7	DTT	3.612	32263	3.562
10	D	4.191	164662	5.065
36	E	8.637	3885593	195.364
39	N	10.119	126689	5.823
47	T	11.800	4253315	221.795
49	G	12.794	6554707	358.310
51	DMPTU	13.301	1304303	360.121
55	H	13.890	1979396	86.625
58	A	14.504	2657689	107.889
60	Y	15.256	2531152	97.092
76	M	19.375	2305937	85.081
77	V	19.633	1484130	51.165
79	DPTU	20.209	2596111	
82	W	20.859	616855	14.667
84	K	21.283	4217974	113.174
86	I	22.370	1744280	57.246
87	L	22.766	2645355	101.287
97	DPU	24.695	969397	
Total			40069806	

Data File	: 10048_09-02-2022_D04.lcd
Sample Name	: Sumit Mukherjee, Sample 1
Method File	: 10048_09-02-2022.lcm
Background Data File	: 10048_09-02-2022_D03.lcd

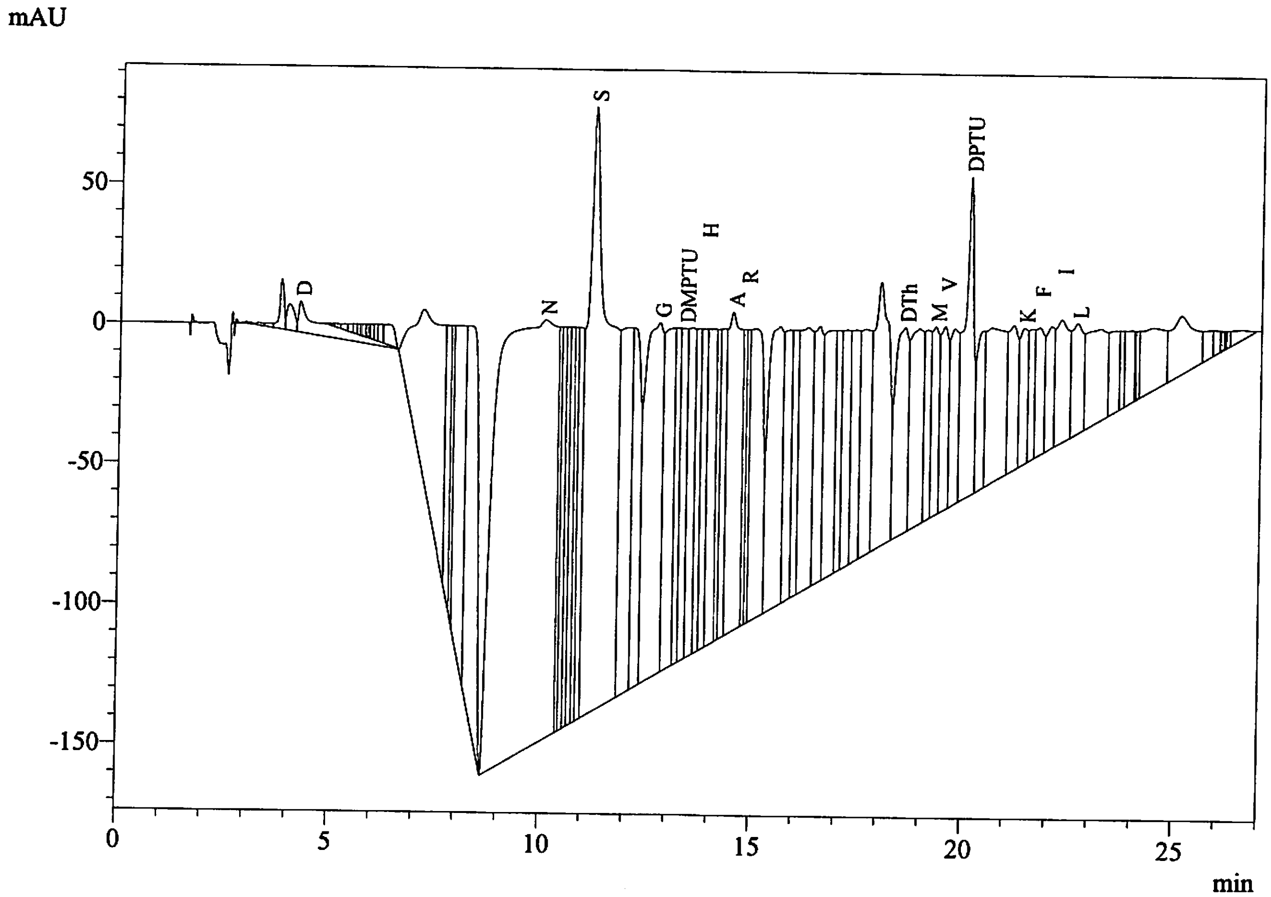

Peak Table
PDA Ch1 269nm

Peak#	Name	Ret. Time	Area	Conc.
10	D	4.253	472680	14.538
32	N	10.057	14487144	665.842
39	S	11.214	7806437	431.171
42	G	12.768	3621306	197.957
45	DMPTU	13.356	1175989	324.693
48	H	13.854	1060557	46.414
52	A	14.510	2560547	103.945
53	R	14.816	541185	27.714
67	DTh	18.586	1566119	176.499
70	M	19.305	783938	28.925
71	V	19.529	857000	29.545
73	DPTU	20.116	1699329	
77	K	21.405	626729	16.816
79	F	21.770	629053	21.285
81	I	22.287	934942	30.684
82	L	22.662	747197	28.609
Total			39570152	
