# Supplementary figures and images for: Activation of the Plasmodium Egress Effector Subtilisin-Like Protease 1 Is Mediated by Plasmepsin X Destruction of the Prodomain
Source: mBio. 2023 Apr 10;14(2):e00673-23. doi: 10.1128/mbio.00673-23 (PMC10128010; doi:10.1128/mbio.00673-23)

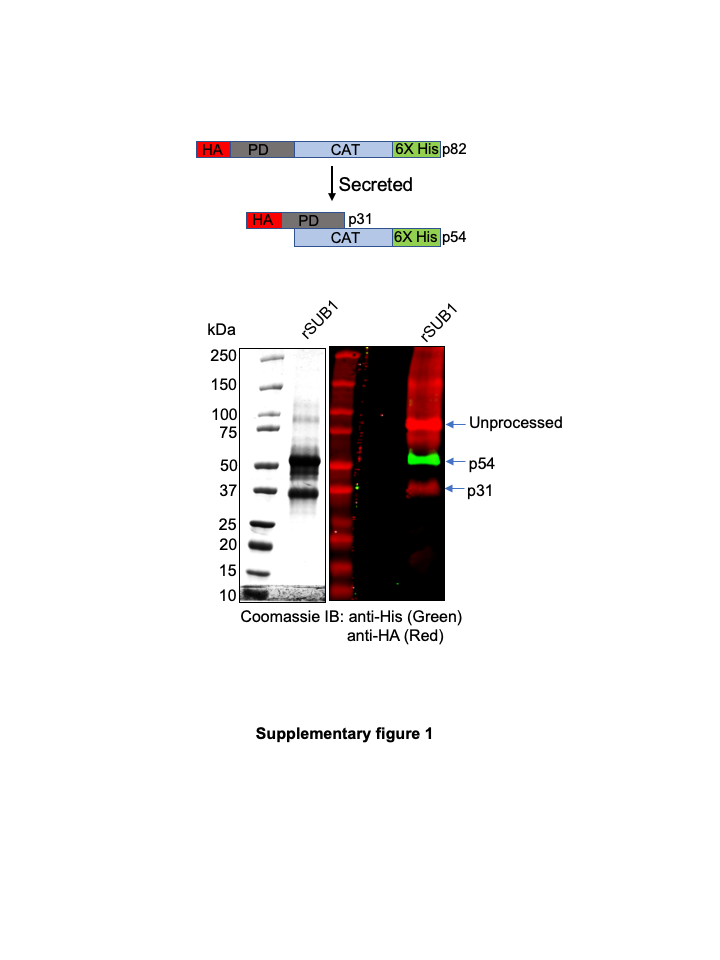

Supplement: FIG S1 [file mbio.00673-23-s0001.tif]

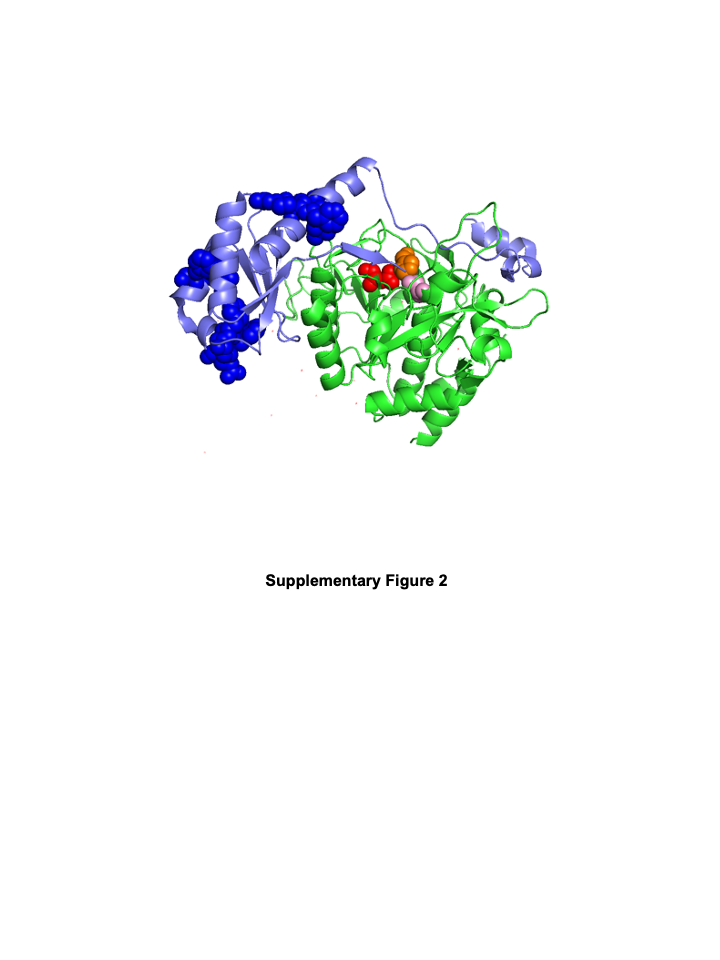

Supplement: FIG S2 [file mbio.00673-23-s0002.tif]

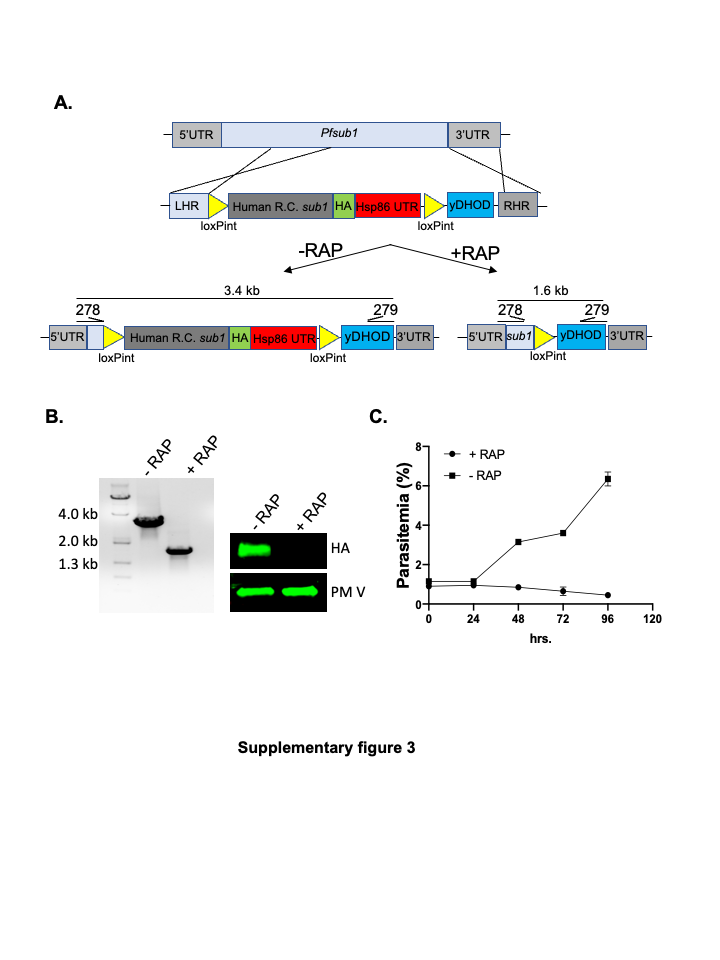

Supplement: FIG S3 [file mbio.00673-23-s0003.tif]

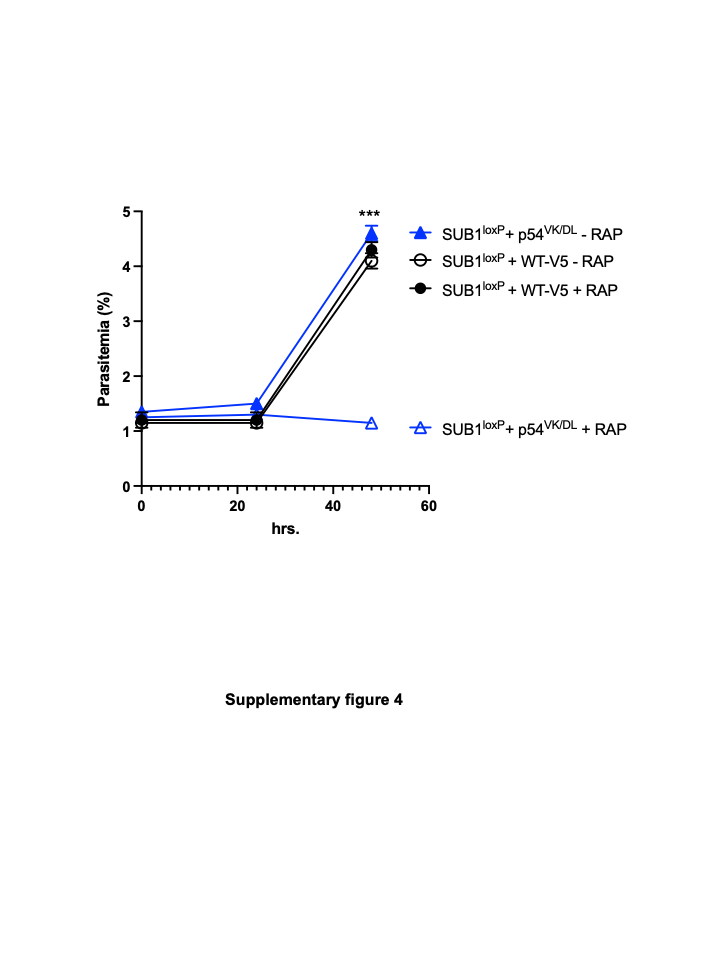

Supplement: FIG S4 [file mbio.00673-23-s0004.tif]

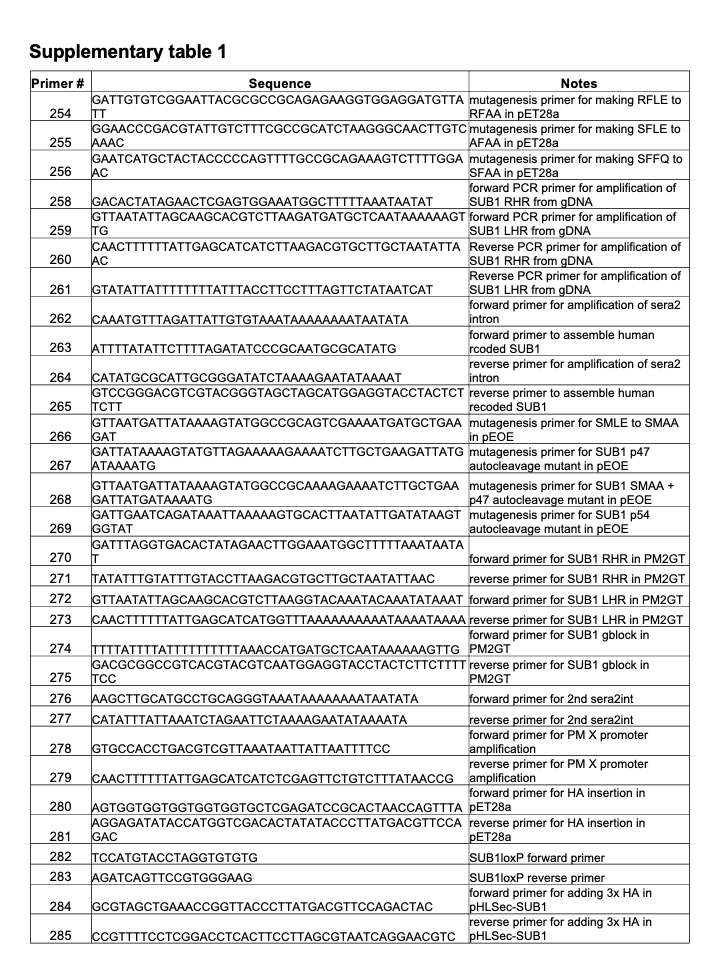

Supplement: TABLE S1 [file mbio.00673-23-s0005.tif]
